# Post-transcriptional air pollution oxidation to the cholesterol biosynthesis pathway promotes pulmonary stress phenotypes

**DOI:** 10.1038/s42003-020-01118-6

**Published:** 2020-07-22

**Authors:** Juan C. Gonzalez-Rivera, Kevin C. Baldridge, Dongyu S. Wang, Kanan Patel, Jamie C. L. Chuvalo-Abraham, Lea Hildebrandt Ruiz, Lydia M. Contreras

**Affiliations:** McKetta Department of Chemical Engineering, University of Texas at Austin, 200 East Dean Keeton Street, Stop C0400, Austin, TX 78712 USA

**Keywords:** High-throughput screening, Transcriptomics, RNA

## Abstract

The impact of environmentally-induced chemical changes in RNA has been fairly unexplored. Air pollution induces oxidative modifications such as 8-oxo-7,8-dihydroguanine (8-oxoG) in RNAs of lung cells, which could be associated with premature lung dysfunction. We develop a method for 8-oxoG profiling using immunocapturing and RNA sequencing. We find 42 oxidized transcripts in bronchial epithelial BEAS-2B cells exposed to two air pollution mixtures that recreate urban atmospheres. We show that the FDFT1 transcript in the cholesterol biosynthesis pathway is susceptible to air pollution-induced oxidation. This process leads to decreased transcript and protein expression of FDFT1, and reduced cholesterol synthesis in cells exposed to air pollution. Knockdown of FDFT1 replicates alterations seen in air pollution exposure such as transformed cell size and suppressed cytoskeleton organization. Our results argue of a possible novel biomarker and of an unseen mechanism by which air pollution selectively modifies key metabolic-related transcripts facilitating cell phenotypes in bronchial dysfunction.

## Abstract (web summary)

Gonzales-Rivera et al. develop a method for 8-oxoG profiling using immunocapturing and RNA sequencing. They show that the FDFT1 transcript is susceptible to air pollution-induced oxidation, after identifying 42 transcripts that are differentially oxidized in bronchial epithelial BEAS-2B cells under air pollution conditions relative to clean air. FDFT1 oxidation affects cholesterol synthesis pathway, leading to phenotypes associated with several lung diseases.

## Introduction

Ambient air pollution poses a risk for the development of a broad range of health conditions, predominantly to the cardiovascular and pulmonary systems. Components of air pollution can generate strong oxidizing agents that may induce reactive oxygen and nitrogen species (ROS/RNS) in the lung environment leading to oxidative stress^1^. Among the various effects that are attributed to oxidative stress within the lungs, early alterations in the structure of airway cells and pro-inflammatory cellular events contribute to the premature and progressive decline of pulmonary function^2^. These events are strongly suspected to result in widespread chronic conditions, such as asthma, chronic obstructive pulmonary disease (COPD), and pulmonary fibrosis^3^, predominantly found among the most susceptible population to air pollution affecting hundreds of millions of people worldwide^4^.

The airway epithelium is a critical immunological and protective barrier against airborne environmental insults. It is well documented that exposing pulmonary epithelial cells to harmful gases, particulate matter, or allergens results in morphological phenotypes such as changes in cell adhesion and cell contractility, and presence of multilamellar bodies, microvilli, and vesicles that are characteristic of airway wall remodeling in chronic lung diseases^5,6^. These events underlie a known orchestrated response of signaling factors, membrane receptors, and regulatory molecules^7–9^. Despite efforts to understand the effects of environmental challenges, there is still an enormous potential for the discovery of molecular mechanisms associated with the decline of lung function. This knowledge is critical for developing effective therapeutic strategies.

Advances in gene expression profiling have proved crucial for identifying potential genes and mechanisms implicated in response to toxic exposures^10^. The ability to evaluate the genome and transcriptome has contributed to a better understanding of the mutagenic effects of oxidative stress induced by air pollution; specifically related to DNA damage and reduced DNA repair capacity^11,12^. Unlike oxidative DNA lesions, RNA oxidation has not raised much attention until recently, despite differences in structure and localization that favor the susceptibility of RNAs to chemical modifications by environmental challenges^13^. Among the numerous chemical modifications of RNA molecules (the epitranscriptome), modification of guanine (G) in the form of 8-oxo-7,8-dihydroguanine (8-oxoG) has been the most notable base oxidation in RNA concerning alterations in genetic information^14^. Evidence suggests that the formation of 8-oxoG is not random, as selective RNA transcripts are more susceptible to oxidation regardless of their expression levels^15^. In addition to oxidation post transcription, free guanosines are vulnerable to ROS, and thus free 8-oxoG could be potentially incorporated during transcription^14^. While cells contain mechanisms that degrade 8-oxoG ribonucleotides before RNA synthesis^16^, mechanisms that repair 8-oxoG post synthesis are not known.

Recent studies within environmental health science are contributing to our broader understanding of the impacts of environmental exposures on the epitranscriptome. Air pollution and cigarette smoke have been shown to cause accumulation of 8-oxoG RNA in epithelial A549 cells^17^ and mouse lungs^18^, respectively. This is clinically relevant given that the abnormal accumulation of 8-oxoG modifications of RNAs has been shown in numerous diseases, including early neurodegeneration processes^19^ and severe COPD^18^, suggesting a potential role of 8-oxoG in pathogenesis^20^.

Although 8-oxoG is a valuable biomarker in epidemiologic studies of environmental exposure and related diseases^21,22^, there is little understanding as to how specific 8-oxoG marks could compromise relevant cellular pathways as a result of air pollution. The underlying importance of environmentally induced chemical changes in RNA is relatively unexplored, making the field of environmental epitranscriptomics a widely open research area. In this work, we developed the 8-oxoG RIP-seq method to explore the potential impact of air pollution exposures in chemical oxidation of RNA and we provide a further mechanistic investigation of one of the perturbed pathways resulting from air pollution oxidation. As such, this study contributes to the understanding of how air pollution exposures impact the epitranscriptome, as well as identifying responsive mechanisms and exposure biomarkers that are relevant in diagnostics and therapeutics of diseases.

## Results

### Characterization of cell exposure to air pollution mixtures

Most experimental environmental studies have focused on investigating exposure to a single chemical^23,24^, but in reality, we are continuously exposed to heterogeneous mixtures of agents. For example, in urban areas, air contains oxides of nitrogen and sulfur, ozone, organic compounds, particulate matter, and more. Studying complex mixtures is more biologically relevant because the detrimental effects of exposures involving multiple molecules are much greater than those provided by individual molecules^17^.

Given that the bronchus might experience the highest particle exposures in the lungs^25^, we used bronchial epithelial BEAS-2B cells, a well-established model for toxicity studies^26,27^. It is worth noting that several cell-based models have been established to study respiratory toxicology^28^. Despite not fully capturing the dynamics of the respiratory system, lung cell lines provide a first approximation to understanding transcriptional regulation processes during environmental stress^29,30^ that can be further explored in more complex systems.

**Fig. 1:**
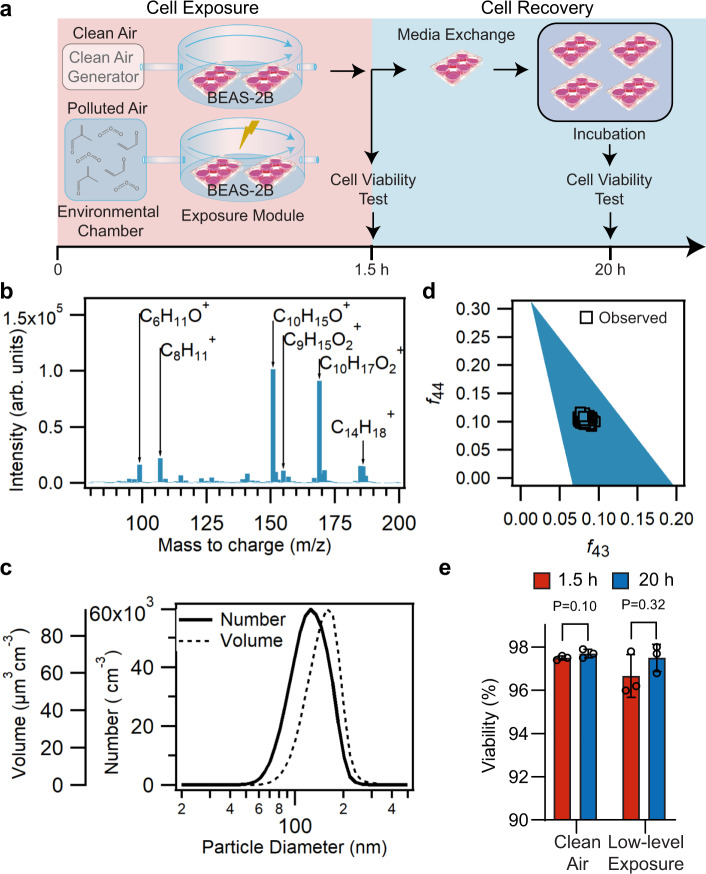
Physicochemical and cell viability characterization of the air pollution mixture derived from low-level VOCs + O_3_ mixture. Concentrations of the initial precursors are shown in Table 1. **a** Schematic of the exposure experiment. Cells are exposed for 1.5 h to the low-level or high-level air pollution mixtures. Cell viability from two cell inserts in a six-well plate is analyzed after exposure, and the remaining inserts are exchanged with fresh media and incubated at 37 °C. After 20 h from starting the exposure, two inserts are analyzed for cell viability. **b** Representative gas-phase composition during one of the low-level exposures (0–1.5 h), measured using the (H_2_O)_n_H_3_O^+^ chemical ionization mass spectrometer (CIMS). Average integrated unit-mass ion intensities are shown. Labels indicate select dominant ions observed at the corresponding m/z. Ions ranging between m/z 2–79 and 201–400 were monitored, but not shown. The integrated ion intensities shown are not adjusted for sensitivities due to lack of authentic standards for oxidation products. **c** Size distribution of secondary organic aerosol as observed by the scanning electrical mobility system (SEMS), averaged over the period between 0 and 1.5 h from the start of the low-level exposure. Lognormal distributions are shown. **d** Typical *f*_44_ vs *f*_43_ profile, an estimator for aerosol oxidation state, observed by the aerosol chemical speciation monitor (ACSM) during the low-level exposure, period (0–1.5 h). Ambient data typically lies within the triangular region. **e** Cell viability at low-level exposures. Percentage of viable cells (at *t* = 1.5 h) after trypsinization of the adhered cells in the inserts, and after cell recovery (*t* = 20 h) determined by trypan blue dye exclusion method in an automatic viability analyzer (Vi-CELL) (*n* = 3 independent experiments). Statistical difference was computed using *t* test analysis (one-tailed homoscedastic). Error bars are expressed as one standard deviation (s.d.).

Here, we exposed BEAS-2B cells to two different mixtures of airborne pollutants for 1.5 h using an air–liquid interface system (Fig. 1a). These mixtures were derived from the reaction of low and high concentrations of acrolein, methacrolein, α-pinene, and ozone (O_3_) in a 10-m^3^ Teflon environmental chamber at 37.3 °C (Supplementary Fig. 1). The initial concentrations of the precursors are summarized in Table 1. These reactions were monitored using a scanning electrical mobility system (SEMS, for monitoring of the particle matter size), an aerosol chemical speciation monitor (ACSM, for monitoring the particle-phase bulk composition), and a high-resolution time-of-flight chemical ionization mass spectrometer (CIMS, for monitoring the molecular composition of the gas phase), collectively confirming that the resulting air mixture composition was similar within independent replicates for each low and high condition.

**Table 1:** Summary of initial precursor concentrations and SOA formed.

Mixture	Replica	Initial concentration of precursors (ppb)				Maximum formed SOA (µg m^−3^)
		O_3_	Methacrolein	Acrolein	α-pinene	
Low level	1	109	97	100	44	50
	2	109	97	100	44	50
	3	100	97	100	44	40
High level	1	3900	670	790	0	60
	2	3700	670	790	0	50
	3	3900	670	790	0	N/A

*N/A* data not available for this exposure, *SOA* secondary volatile aerosol.

It is expected that acrolein, methacrolein, and α-pinene will react to form a combination of substances more reflective of what pulmonary cells might experience in a polluted environment. In this model, acrolein, methacrolein, and α-pinene are volatile organic compounds (VOCs) that act as precursors forming secondary organic aerosol (SOA) by gas-phase reactions with O_3_ and partitioning of the low-vapor-pressure products to the particulate phase. Acrolein and methacrolein are common VOCs found in urban atmospheres, mostly emitted in combustion processes, including tobacco smoke, cooking fumes, forest fires, and combustion of diesel^31,32^, and they are medically relevant because they exacerbate asthma and COPD^33,34^ by mechanisms not fully understood. Furthermore, α-pinene, an abundant monoterpene, is emitted in vast quantities to the atmosphere by vegetation (e.g., by many coniferous trees, such as pine) and it is an important atmospheric precursor of SOA^35^. Lastly, O_3_ is an abundant atmospheric oxidizer associated with oxidative damage to the lungs^36^. We injected concentrations of VOC precursors and O_3_ to form a multi-component gas-phase mixture, including oxidation products such as aldehydes and ketones^37^, which commonly contribute to smog in urban atmospheres^38^. The precursors undergo several generations of chemical reactions that transformed them into SOA^39^ (Fig. 1b; Supplementary Fig. 2a). In this study, the BEAS-2B cells were exposed to a mix of reaction products and unreacted precursors.

The SOA concentration generated in the chamber ranges from ~40–60 μg m^−3^ with particle mode diameter around 100–130 nm (Fig. 1c; Supplementary Fig. 2b). This concentration of airborne fine particles (PM_2.5_—particle diameter < 2.5 μm) corresponds to conditions referred as “unhealthy for sensitive groups” according to National Ambient Air Quality Standards (NAAQs, 1997). Yet, these conditions are typical of moderately polluted megacities^40,41^, during wildfire periods in urban areas in California^42^ or while inside an office building in a U.S. city^43^. We plotted the *f*_44_ vs *f*_43_ triangle profile, an estimator of aerosol oxidation state, obtained from the ACSM during the exposure period of 1.5 hours (Fig. 1d; Supplementary Fig. 2c). Higher *f*_44_ values are associated with greater contribution to the aerosol mass by more oxidized compounds (e.g., doubly oxidized compounds), whereas higher *f*_43_ values are associated with greater contribution to the aerosol mass by lightly oxidized compounds (e.g., singly oxidized compounds). The aerosols generated with the low and high concentration of VOCs + O_3_ precursors fall within the typical range observed in ambient organic aerosol samples (represented by the shaded triangular region in Fig. 1d and Supplementary Fig. 2c, pollution mixtures with low or high oxidative potential, respectively)^44^. Moreover, the aerosols from the low precursor levels have a lower oxidative potential than the aerosols from the high precursor levels; this is evident by comparing the distance of the data points to the superior edge of the triangle in Fig. 1d and in Supplementary Fig. 2b. For simplicity, we will refer throughout the paper to the two concentration levels as low-level and high-level pollution mixture, respectively.

### Minimal damage in cells exposed to the low-level mixture

We first studied changes in cell viability caused by the low-level pollution mixture using the trypan blue exclusion method. As seen in Fig. 1e, cell viability does not significantly increase after exposure to air pollution relative to clean air control cells (*t* test analysis, one-tailed homoscedastic, *P*-value > 0.05). Moreover, most of the cells remained viable after 20 h, indicating that the low-level pollution exposure is nonlethal. We also evaluated the cytotoxicity of the low-level pollution exposure using the enzymatic activity of lactate dehydrogenase (LDH), revealing comparable levels of LDH between exposed cells and controls immediately after exposure (*t* = 1.5 h) (Supplementary Fig. 3).

We conducted transcriptomics analysis to compare expression changes when cells were exposed to the low-level pollution mixture relative to clean air controls. Because the cells exposed in this study have not shown signs of mortality, observed transcriptomics patterns are considered indicative of cell responses and not reflective of major cellular changes due to programmed cell death. The transcriptomics analysis shows differential expression of 881 mRNA transcripts with an adjusted *P*-value < 0.05. Of these, 336 transcripts exhibit increased expression with a fold change >2, and 545 transcripts exhibit decreased expression with fold change <0.5 (Supplementary Fig. 4a, Supplementary Data 1, 2).

The pathway analysis highlights that many upregulated transcripts are linked to pyruvate metabolism (Supplementary Fig. 4b), a critical component of integrated metabolic processes (i.e., glycolysis and acetyl-coA production) necessary for cell survival. Increased pyruvate metabolism has been shown to promote fibrotic responses^45^, acute exacerbation of asthma^46^, and non-small-cell lung cancer^47^. Moreover, lipid synthesis necessary for maintaining cellular membranes is dependent on acetyl-CoA—produced by pyruvate metabolism—, thus indicating a link between cellular membrane integrity and cellular metabolism. Specifically, we identify transcripts belonging to classes of adhesive contacts through the interaction of transmembrane proteins (i.e., adherens junctions and focal adhesion) as differentially expressed, which are essential to maintain the protective epithelial barrier against environmental insults and pathogenic infection^48,49^.

The connection between metabolic pathways, cell junctions, and poor air quality revealed by this dataset is consistent with previous studies in BEAS-2B cells, which established alterations in cellular metabolic processes^50,51^. In these earlier studies, cells were subjected to submerged exposure of PM2.5 at 10 μg cm^−2^ of cell culture and 50 μg ml^−1^, which are equivalent to ~15 and ~22 times our particle exposure dose, respectively. Moreover, in vivo studies evaluating gene expression in mice revealed comparable alterations in gene expression of cell–cell adhesion and calcium transport pathways after doses of 300 μg of PM2.5 (equivalent to ~44 times our particle exposure dose)^52^. These results support our cell exposures, and confirm that specific patterns of transcriptional changes can be recognized from air pollution exposure.

### Increased RNA oxidation in cells exposed to air pollution

**Fig. 2:**
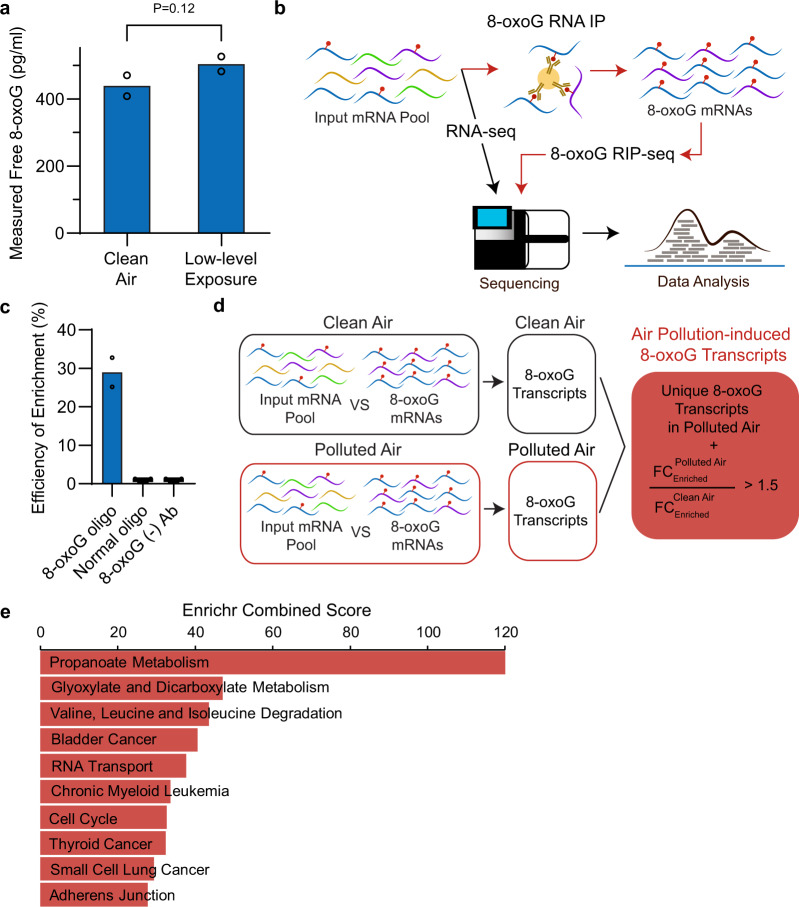
8-oxoG-RIP sequencing shows that certain mRNAs are more prone to oxidation by air pollution. **a** Free 8-oxoG nucleosides from the total RNA were quantified shortly after low-level exposure (*t* = 1.5 h) by ELISA (*n* = 2 independent experiments). These levels are equivalent to 1.46 ± 0.10 nM (or 65.9 ± 4.69 pg of 8-oxoG/µg of RNA) and 1.68 ± 0.07 nM (or 75.6 ± 3.30 pg of 8-oxoG/µg of RNA) in the control and exposed cells, respectively. **b** Schematic of the 8-oxoG-RIP-seq approach. Briefly, RNA is extracted and depleted of rRNA in BEAS-2B cells exposed for 1.5 h to air pollution mixtures or clean air. A fraction of the resulting pool of RNA is immunoprecipitated (RIP) in the presence of an antibody that selectively binds 8-oxoG-containing RNAs. Then, RNA library preparation and sequencing are performed in the unenriched mRNA fraction (pool before the RIP step) and the 8-oxoG mRNA enriched pool (after the RIP step). **c** Enrichment of P^32^-labeled 8-oxoG oligomers using immunoprecipitation (IP) compared with normal oligomers determined by scintillator. As a negative control, 8-oxoG oligomers were incubated without the presence of anti-8-oxoG antibody (*n* = 2 independent experiments). **d** Schematic of the methodology used to identify air pollution-induced 8-oxoG transcripts. 8-oxoG-enriched transcripts from each condition were identified by comparing the 8-oxoG RIP mRNA relative to the input mRNA pools. Then, the resulting 8-oxoG-enriched transcripts were compared between exposure conditions to identify air pollution-induced 8-oxoG transcripts, which include unique 8-oxoG-enriched transcripts in the air pollution pool or 8-oxoG-enriched transcripts present in both the air pollution mixture and control exposures that exhibited a fold-change (FC) ratio (exposure to control) >1.5. **e** KEGG pathway analysis for air pollution-induced 8-oxoG transcripts in cells exposed at the low-level mixture for 1.5 h. This data set is presented in Supplementary Data 4. Statistical difference was computed using *t* test analysis (one-tailed homoscedastic).

To assess the extent of RNA oxidation in BEAS-2B cells exposed to air pollution mixtures, we measured concentrations of 8-oxoG ribonucleotides using ELISA, a technique that has been widely used to detect guanine oxidations in RNA and DNA^17,53,54^. Cells exposed to the low-level pollution mixture exhibited slightly higher levels of 8-oxoG as compared with control cells (Fig. 2a). Even though the effect did not reach statistical significance (*t* test analysis, one-tailed homoscedastic, *P*-value = 0.12), the levels measured are consistent with 8-oxoG concentrations reported in human and animal samples^55–57^. When cells were exposed to the high-level pollution mixture, we observed a significant increase in 8-oxoG levels relative to clean air controls (Supplementary Fig. 5), consistent with the high oxidative state of the particle phase (Supplementary Fig. 2c). These results suggest that air pollution causes physiologically relevant levels of RNA oxidation in lung cells during a short exposure period of 1.5 h (relative to the cell line’s doubling time of ~24 h). Likewise, studies in heart mouse tissue have detected changes in RNA oxidation after 1 h of inducing oxidative stress by decreasing oxygen tension^58^. Furthermore, ex vivo exposure of the total RNA (extracted from BEAS-2B before exposure) supports that the chemical components in the air pollution mixtures could directly influence RNA oxidation (Supplementary Fig. 6).

It is worth noting that we measured moderated levels of basal RNA oxidation in the clean air controls. Previous evidence suggests that even in the absence of exogenous agents, endogenous cellular processes generate ROS that may not pose a functional burden to the cell^59,60^. Indeed, ROS can act as important signaling molecules in some cases, i.e., angiogenesis^61^ and inflammation^62^. Although some level of basal oxidation is expected and could play functional roles as epitranscriptomics marks^63^, this specific phenomenon requires further investigation in future work.

### 8-oxoG RIP-seq enables detection of oxidation after exposure

We developed an integrated RNA immunoprecipitation (RIP) assay of 8-oxoG with RNA sequencing (8-oxoG RIP-seq) to identify which RNA transcripts are more susceptible to oxidation by air pollution (Fig. 2b). Given that the process of RNA oxidation is selective^15^, we expect to identify cellular pathways enriched in oxidized transcripts as indicative of targeted susceptibility by air pollution-induced oxidation. We employed an anti-8-oxoG antibody (clone 15A3) that can recognize 8-oxoG in both DNA and RNA^15,58,64,65^, and has been used for 8-oxoG immunoprecipitation of miRNA and mRNAs^15,58^.

We conducted Dot blotting to characterize the cross-reactivity of the selected antibody to 8-oxoG over common methylated and oxidized RNA modifications (N6-methyladenine (m^6^A), 8-oxo-7,8-dihydroadenine (8-oxoA), 5-hydroxycytosine (ho^5^C), 5-hydroxyuracil (ho^5^U), and 5-formylcytosine (f^5^C)), as well as unmodified G (Supplementary Fig. 7a). Our results show that the antibody used is highly specific to 8-oxoG-marked RNAs (particularly when marked internally) relative to nonmodified RNAs and RNAs marked with other modifications (i.e., 8-oxoA, ho^5^C, ho^5^U, f^5^C, and m^6^A were tested). Given the lack of signal in the 10-mer containing one 8-oxoG modification at the second position from the 5′ end, the antibody may fail to capture 8-oxoG-modified RNAs near the 5′ end. Our results also indicate little sequence bias, observed by the linear behavior between the number of 8-oxoG marks and the binding signal (Supplementary Fig. 7b). We then immunoprecipitated radiolabeled 8-oxoG-containing oligos to monitor the IP efficiency by scintillation (Fig. 2c). We used unmodified oligos and incubations in the absence of the antibody as negative controls. Based on our 8-oxoG immunoprecipitation, we found an efficiency of ~30%. In contrast, other known 8-oxoG immunoprecipitation approaches have reported lower efficiencies of ~8%^66^. These low efficiencies on 8-oxoG immunoprecipitation could reflect a structure-dependent bias, which has been reported in other RNA modification antibodies^66,67^.

To discriminate 8-oxoG resulting from air pollution exposure from native cellular 8-oxoG and artifactual oxidation that might be caused during sample preparation^68^, we incorporated the statistical comparisons shown in Fig. 2d. Briefly, we first identified 8-oxoG enrichment within the mRNA pool in either exposed or control cells. At this step, we compared the distribution of each transcript between the pool of transcripts that bound to the 8-oxoG-specific antibody and the input pool (the total RNA pool in the absence of 8-oxoG antibody immunoprecipitation) in either exposed or control cells (with an adjusted *P*-value < 0.1, and fold change >2). Then, we compared the resulting groups of 8-oxoG transcripts in the exposed cells and the control cells to discriminate transcripts that are either (A) uniquely represented in the air pollution group or (B) that although present in both exposed and control pools, are at least 1.5 times more abundant in exposed cells. The resulting group, referred to as air pollution-induced 8-oxoG transcripts, has a minimum log2-fold-change enrichment of 6.7 (Supplementary Data 3 and Supplementary Fig. 8), a threshold sufficiently high to confidently assume that this analysis removed background noise generated from nonspecific interactions (between mRNA transcripts and protein A magnetic beads or 8-oxoG antibody) or random artifactual oxidation.

This analysis yielded 707 transcripts enriched in 8-oxoG modifications in BEAS-2B cells after 1.5 h of the low-level air pollution exposure (Supplementary Data 3). Previous studies have identified ~3400 oxidized transcripts in mice expressing familial amyotrophic lateral sclerosis (ALS)-linked SOD1 mutant^69^ and ~2400 oxidized transcripts in *Saccharomyces cerevisiae* treated with H_2_O_2_^70^. However, these studies lack statistical analyses to distinguish between basal oxidation and specific oxidation induced by the treatment condition. Furthermore, we followed recommendations to prevent artificial oxidation of the RNA during sample preparation, including the use of O_2_-depleted solutions and avoiding RNA fragmentation before immunoprecipitation^68^.

According to the analysis of KEGG pathways in Enrichr^71^, the 707 oxidized transcripts by air pollution are involved in carbohydrate and amino acid metabolism (i.e., propanoate metabolism, glyoxylate and dicarboxylate metabolism, and valine, leucine, and isoleucine degradation) cancer pathways (i.e., bladder cancer, chronic myeloid leukemia, thyroid cancer, and small-cell lung cancer), RNA transportation, and adherens junction (protein bridges connecting actin cytoskeleton in neighboring cells) (Fig. 2e; Supplementary Data 4). Multiple evidence supports that metabolic reprograming occurs during lung pathogenesis, especially during chronic pulmonary diseases. This type of metabolic alteration has been observed in the acetyl-CoA pathway (i.e., pyruvate metabolism, previously identified as enriched in upregulated transcripts) and in the biosynthesis of amino acids and lipids^72,73^. For instance, transcripts and proteins in the propanoate pathway as well as in the valine, leucine, and isoleucine-degradation pathway are altered in COPD rat models^74^. Of relevance to this study, the propanoate pathway and the valine, leucine, and isoleucine pathway share five transcripts that were identified in our oxidative enrichment analysis (Supplementary Data 3), including the trifunctional enzyme subunit alpha, mitochondrial (*HADHA*), 2-oxoisovalerate dehydrogenase subunit alpha, mitochondrial (*BCKDHA*), propionyl-CoA carboxylase alpha chain, mitochondrial (*PCCA*), peroxisomal bifunctional enzyme (*EHHADH*), and ethylmalonyl-CoA decarboxylase (*ECHDC1*).

We found an accumulation of 8-oxoG in RNA transcripts associated with adherens junctions (Fig. 2e). Of interest, afadin (*AFDN*) and TGF-beta receptor type-1 (*TGFBR1*) are also involved in acute myeloid leukemia (also enriched in oxidized transcripts). Likewise, the receptor tyrosine-protein kinase erbB-2 (*ERBB2*) is both an essential component in the regulation of adherens junctions and it is a well-established therapeutic target in bladder cancer^75^ (enriched in oxidized transcripts). Taken together, these data evidence that RNA oxidation by air pollution may be a relevant physiological process in pathogenesis and/or progression of pulmonary diseases and cancers.

### 8-oxoG is selective and correlates with mRNA downregulation

One important feature of RNA oxidation is that oxidation occurs selectively and independent of the RNA abundance in the cellular pool^15^. Therefore, we investigated the distribution of the 707 oxidized transcripts by calculating the ratio of oxidized transcripts versus all detected transcripts in ten averagely divided expression bins (Supplementary Fig. 9a). Our results show that these transcripts scatter among low- and high-expression bins, suggesting that oxidation occurred regardless of the mRNA expression levels. Other molecular aspects that make certain RNAs more prone to oxidation require further investigation in the literature.

Furthermore, previous studies suggest that 8-oxoG modifications can influence mRNA fate (i.e., by affecting mechanisms of transcriptional regulation, stability, etc.)^53,76,77^. Although we found only 28 oxidized transcripts that were statistically downregulated (Supplementary Fig. 4a; Supplementary Data 5), ~81% of the oxidized transcripts are in the negative fold-change region of the differential expression volcano plot (Supplementary Fig. 9b), indicating that oxidized transcripts are more prone to decrease in expression. While this observation argues that oxidized transcripts are more likely targeted for degradation^53^, many 8-oxoG-modified transcripts could be transcriptionally upregulated at the same time (e.g., to restore the population of turned-over and aberrantly oxidized transcripts). This situation could produce no net change in expression levels which could explain why several oxidized transcripts in our exposures did not reach significant changes in expression levels.

### Cholesterol synthesis is sensitive to air pollution oxidation

To better understand transcriptome patterns that are consistently modified as a result of air pollution, we conducted exposures in BEAS-2B cells using the high-level pollution mixture, previously reported to generate a significant increase in RNA oxidation^17^. Because air pollution exposure is uneven in the bronchiole region, it is expected that certain areas could exhibit up to nine times more stress^25^, which may overwhelm cellular defenses and elicit clear defects in lung epithelial cells. When we exposed BEAS-2B cells to the high-level pollution exposure for 1.5 h, we observed stronger patterns of RNA oxidation and cytotoxicity effects than in the low-level exposure. The high-level mixture induced a significant increase in RNA oxidation relative to clean air samples (Supplementary Fig. 5), in agreement with the high particle-phase oxidative potential of this mixture (Supplementary Fig. 2c). We also detected a slight decrease in the percentage of viable cells, the viability after exposure was ~88% (*t* = 1.5 h) and ~93% at the end of the recovery period (*t* = 20 h) (Supplementary Fig. 10a). Even though both low and high conditions share similar SOA concentrations (~40–60 μg m^−3^) and particle mode diameter (~100–130 nm), these data support that metrics of the oxidative capacity of air pollution (e.g., cellular oxidation of RNA) are predictors of the cytotoxicity effects of air pollution^78^.

**Fig. 3:**
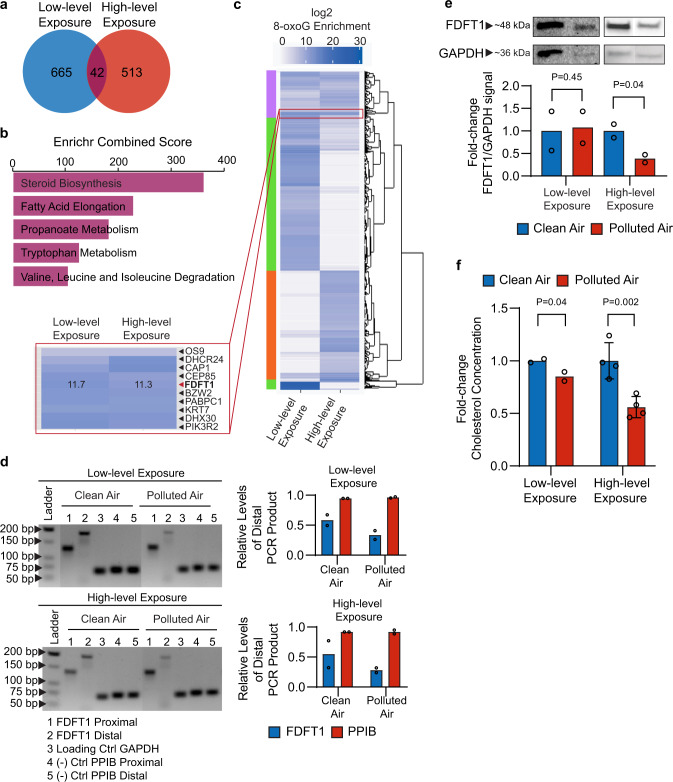
Exposure of BEAS-2B cells to air pollution leads to alterations in cholesterol synthesis. **a** Overlap between the air pollution-induced 8-oxoG transcripts derived from exposure at low-level and the high-level mixtures (Supplementary Data 7). **b** KEGG pathways analysis for the 42 8-oxoG transcripts overlapping between the low-level and high-level air pollution mixtures. **c** Heatmap showing 8-oxoG enrichment of low-level (Supplementary Data 3) and high-level exposures (Supplementary Data 6). Color scale represents 8-oxoG enrichment as log2-fold-change values. Transcripts with similar enrichment were clustered together using ggdendrogram R script. **d** PCR products of FDFT1–215 cDNA synthesized from low-level and high-level exposures. PCR products were separated in 3% agarose gel and stained with ethidium bromide. GAPDH was used as internal normalization, and PPIB was used as a negative control. The amount of PCR product was detected by densitometry using TotalLab CLIQS, and normalized by the level of the internal GAPDH product. The ratio of normalized distal/proximal products are plotted for FDFT1–215 and PPIB (*n* = 2 independent experiments). **e** Western blot of FDFT1 in BEAS-2B cells after exposures to the two air pollution levels (*n* = 2 independent experiments). Detection of GAPDH was used as internal loading control, which showed unchanged expression levels in the transcriptomics analysis. The signal intensity from the bands was quantified by densitometry using TotalLab CLIQS. **f** Endogenous cholesterol measured by a colorimetric assay from whole cellular lysates collected after low-level (*n* = 2 independent experiments) and high-level exposures (*n* = 4 independent experiments). Statistical difference was computed using *t* test analysis (one-tailed homoscedastic). Error bars are expressed as one standard deviation (s.d.).

We applied the 8-oxoG RIP-seq analysis to BEAS-2B cells exposed to the high-level pollution mixture for 1.5 h. Using the statistical analysis described above, we identified 555 transcripts enriched in 8-oxoG modifications in the high-level pollution exposure (Supplementary Data 6). This analysis showed a similar distribution of gene expression consistent with the low-level pollution data (Supplementary Fig. 10b, c). Of these 555 oxidized transcripts, 42 were previously found to be significantly enriched in 8-oxoG at the low-level exposure (Fig. 3a; Supplementary Data 7). The pathway analysis of these 42 transcripts showed that they are mainly involved in steroid biosynthesis, fatty acid elongation, and propanoate metabolism (Fig. 3b). The 8-oxoG-enriched transcripts related to proteins in the steroid biosynthesis pathway are among the most enriched and consistent in oxidation, with two (*FDFT1* and *DHCR24*) out of 19 enzymes involved in this pathway found significantly oxidized under both exposure conditions (Supplementary Data 3, 6).

The heatmap in Fig. 3c illustrates patterns of oxidation in response to the two exposure conditions. Three main clusters indicate patterns of 8-oxoG enrichment among the identified transcripts uniquely oxidized at low-level pollution exposure (green block), high-level pollution exposure (orange block), and commonly oxidized at both exposure conditions (purple block). Of importance, we found a small region of ~10 transcripts in the purple block that are evenly enriched, this region includes the FDFT1 and DHCR24 transcripts.

### FDFT1 is altered by air pollution-prompted oxidation

Little work to date has been done in the literature validating transcriptome patterns in air pollution exposures to different phenotypes, However, a better understanding of the link between transcriptomics changes that result from air pollution exposures and the development of different phenotypes represents a valuable area of investigation in environmental toxicology. To explore the significance of RNA oxidation, we further studied the functional implications of 8-oxoG modifications in the farnesyl-diphosphate farnesyltransferase 1 (FDFT1) transcript, a key regulatory enzyme in the cholesterol biosynthesis pathway. *FDFT1* encodes for a membrane-associated protein, also known as squalene synthase, that has implications in developmental diseases^79^ and cancers^80^.

The FDFT1 transcript detected as oxidized by air pollution, under both exposure conditions (low and high levels), is thought to undergo nonsense-mediated decay (FDFT1–215, Ensembl transcript ID: ENST00000529464), one of the RNA-quality control processes that rely on the recognition of abnormal mRNA by the ribosome^53^. We focused on this transcript, given that we observed its oxidation under both exposure conditions and given its key role in cholesterol biosynthesis, which we hypothesized to be particularly relevant to cytoskeletal properties known to be affected in conditions of lung diseases^81,82^.

Our transcriptomic data show that the FDFT1–215 transcript was downregulated at both low-level and high-level pollution mixture, although this trend has higher statistical significance at the higher oxidative exposure (adjusted *P*-value < 0.05). The levels of oxidized FDFT1–215 transcript after 8-oxoG IP were further verified by the quantification of its copy number using RT-qPCR (Supplementary Fig. 11).

We adapted a reverse transcription truncation assay to validate the oxidation of the FDFT1–215 transcript via an antibody-free approach^83^. We used chemical tagging of 8-oxoG to leave a bulky moiety that induces reverse transcription stops^13,66,84^. In this method, K_2_IrBr_6_ acts as a mild one-electron oxidant that reacts with 8-oxoG—without introducing oxidative modification to G—to form an electrophilic intermediate that can react with a primary-amine nucleophile to yield a stable amine-conjugated product^13,66^.

After reverse transcription of the labeled transcripts, we carried out PCR using primers near the 5′ end (proximal) and the 3′ end (distal). The resulting accumulation of proximal products can be compared with the distribution of distal products to identify oxidized transcripts by gel electrophoresis (Supplementary Fig. 12). We selected the housekeeping proteins glyceraldehyde 3-phosphate dehydrogenase (GAPDH) and peptidyl-prolyl cis–trans isomerase B (PPIB) that remained unaffected by the exposure according to our 8-oxoG RIP-seq data as internal normalization and negative control, respectively. The ratio of distal/proximal FDFT1–215 products represents the relative level of complete FDFT1–215 product relative to that of truncated FDFT1–215 product. The level of 8-oxoG oxidation is determined by a reduction in the ratio from exposed cells as compared with that from the control. The decrease in the relative level of distal PCR product for both exposures indicates oxidation of FDFT1–215 transcript (Fig. 3d). In contrast, the relative levels of distal PPIB were almost identical to the levels of proximal PPIB.

Given that changes in transcriptional stability of 8-oxoG mRNAs may reduce protein expression^53^, we tested FDFT1 levels in protein extracts from BEAS-2B cells exposed to air pollution by western blotting. After normalizing the signal by the GAPDH loading control, FDFT1 expression significantly decrease in the cells exposed to the high-level exposure by 2.5-fold change compared with the control (*t* test analysis, one-tailed homoscedastic, *P*-value < 0.05) (Fig. 3e). At the low-level exposure, FDFT1 levels remain unchanged relative to the clean air control, as expected based on the similar trends observed by transcriptomics data.

Since the FDFT1 protein regulates the first specific step in the cholesterol pathway, we then tested the levels of cholesterol in whole cellular lysates by a colorimetric assay. As seen in Fig. 3f, cholesterol content decrease at both air pollution conditions, and interestingly, the reduction is more significant as the levels of air pollution increased. Overall, our findings suggest that RNA oxidation in the FDFT1–215 transcript accumulates at non-lethal conditions, in a way that alters gene and protein expression and promotes dysregulation of the cholesterol synthesis pathway at increased air pollution concentrations.

### FDFT1 downregulation echoes environmental exposure phenotypes

**Fig. 4:**
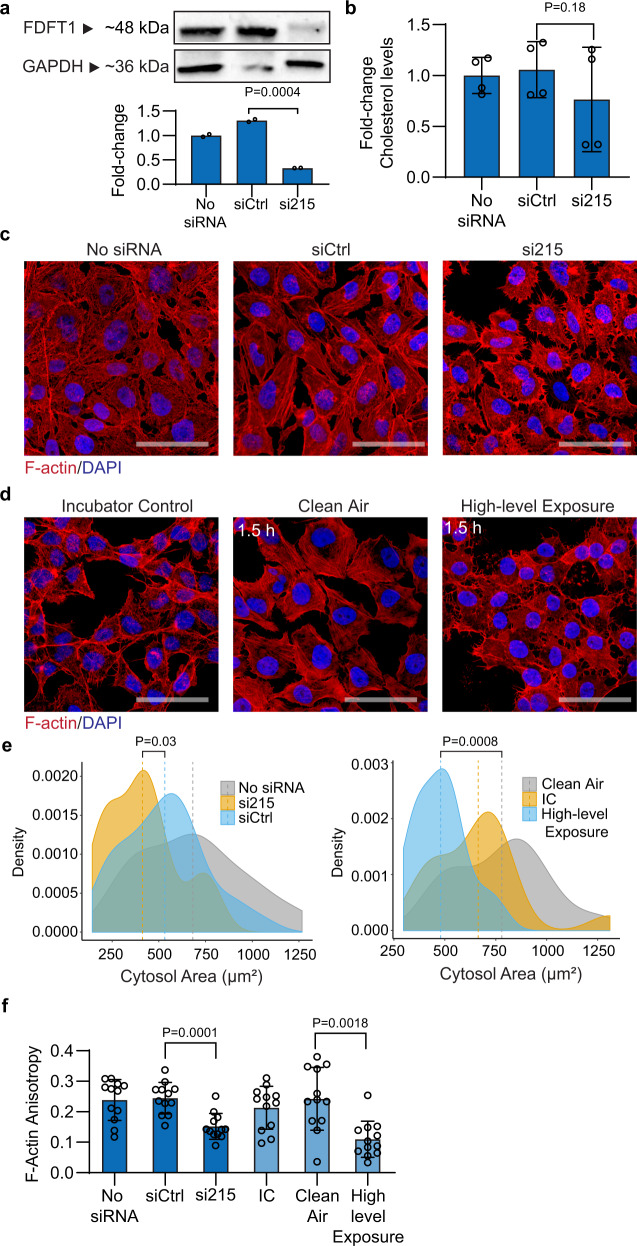
Downregulation of FDFT1 (farnesyl-diphosphate farnesyltransferase 1) in BEAS-2B cells is linked to early alterations induced by air pollution. **a** Western blot analysis of FDFT1 in BEAS-2B cells after 24 h of siRNA antisense knockdown of FDFT1 (*n* = 2 independent experiments). A scrambled sequence siRNA was used as a control (siCtrl). **b** Endogenous intracellular cholesterol in FDFT1 knockdowns of BEAS-2B cells (*n* = 4 independent experiments). **c** Confocal fluorescent microscopy images of F-actin staining with Alexa Fluor 594 phalloidin and nuclei staining with DAPI of BEAS-2B cells using an optical magnification of ×63 (*n* = 2 independent experiments). Scale bar equals 50 µm. The images are representative of two independent FDFT1 knockdown in BEAS-2B cells. **d** Confocal fluorescent microscopy of BEAS-2B cells exposed to the high-level exposure (*n* = 2 independent experiments). **e** F-actin area of FDFT1 knockdown cells and cells exposed to the high-level mixture. Area quantified from at least 15 cells per condition using Fiji Image J. **f** Anisotropy of actin fibrils in FDFT1 knockdown cells and cells exposed to the high-level mixture. Anisotropy was measured using the ImageJ plug-in FibrilTool. An anisotropy score of 0 is given for no order (purely isotropic fibrils), and 1 is given for perfectly parallel fibrils (purely anisotropic arrays). This analysis was conducted in 10 µm × 5 µm regions on ten cells for each condition. Statistical difference was computed by *t* test analysis one-tailed homoscedastic (panels **a**, **b**) and Mann–Whitney test (panels **e**, **f**). Error bars are expressed as one standard deviation (s.d.).

To understand the deleterious effects of downregulation of FDFT1 on cellular function, we knocked down FDFT1 in BEAS-2B cells using small interfering RNA (siRNA). We designed a siRNA to target the FDFT1–215 transcript (referred to here as si215). As negative controls, we used a scrambled sequence siRNA control (predesigned silencer select negative control sequence No. 1, Thermo Fisher Scientific) and siRNA-untreated cells. We confirmed decay in FDFT1 protein levels in the silenced cells by western blotting (Fig. 4a), with a transfection efficacy of at least 70%. Moreover, we observed a decrease in cellular cholesterol after 24 h of si215 treatment (Fig. 4b). Because cholesterol is critical in cellular membranes for fluidity, stiffness, and structural support of cytoskeleton^85^, we inspected the effect of defective cholesterol synthesis (driven by FDFT1–215 knockdown) on cell morphology. We observed that FDFT1 knockdown leads to substantial morphological alterations in BEAS-2B cells, including alterations in cellular shape and retraction of cell size (Fig. 4c). Yet, consistent with the fact that cholesterol is a key regulator of membrane and actin cytoskeleton organization^86^, si215 cells experienced drastic changes in F-actin integrity and membrane ruffling, as well as gap formations between adjacent cells. Notably, these morphological changes are detected without considerable alterations in cell viability (Supplementary Fig. 13).

To test the potential association of the observed defective cell morphology phenotype stimulated by FDFT1 knockdown and air pollution exposure, we then analyzed the morphological changes in BEAS-2B cells after high-level pollution exposure for 1.5 h. Strikingly, the knockdown of FDFT1 reproduced the phenotypic alterations spontaneously occurring during air pollution exposure (Fig. 4c, d). As such, the si215 cells suffered a significant retraction of ~22% in the cytosol area compared with siCtrl cells (Fig. 4e), as well as substantial membrane ruffling, suggesting loss of cell adhesion. This effect results in loss of cell-to-cell contacts as evidenced by the formation of distinct intercellular gaps (Fig. 4c). Similarly, exposed cells acquired heterogeneous shapes and experienced significant irregular retraction of the cytosol area by 28%, whereas clean air control cells maintained their original epithelial-like morphology. Furthermore, we analyzed the heterogeneity in cortical actin filament orientations (or F-actin anisotropy—an estimator of microfilament organization) using FibrilTool^87^. In si215-silenced cells, the anisotropy score significantly decreased by ~38% relative to the siCtrl cells. Likewise, we observed significant rearrangements in actin filaments post exposure with an averaged decrease of ~55% in the anisotropy score (Fig. 4f). Notably, key biological processes highly dependent of the cytoskeleton and cell-to-cell adhesion (e.g., adherens junction and cell adhesion) were found to be extensively impacted by the air pollution conditions in our 8-oxoG transcriptional and functional analysis (Fig. 2e; Supplementary Data 4, 8). Such alterations in the actin microfilaments networks have been described to mediate pro-inflammatory signals^88^, which could contribute to inflammatory responses in early stages of chronic pulmonary diseases by inhalation of environmental pollutants^89^. This inflammatory contribution to lung dysfunction is beyond the scope of this study.

Importantly, the observed morphological phenotypes in BEAS-2B are consistent with previous air exposure studies in cultured pulmonary cells. Cigarette smoke exposures have been described to reduce F-actin content and promote intracellular gap formation in both bovine pulmonary artery endothelial cells and primary alveolar type II epithelial cells^90^. Likewise, studies using urban PM2.5 with a dose of 10 µg cm^−2^ of cell culture area for 24 h, and radical-containing PM10 (particles with diameter <10 µm) with a dose of 20 µg cm^−2^ of cell culture area for up to 24 h have been reported to prompt microfilament rearrangements and incomplete cell-to-cell contact in BEAS-2B cells^51,91^.

Taken together, our data indicate that loss of FDFT1, acting in the cholesterol biosynthesis, leads to morphological phenotypes in airway cells associated with air pollution exposure. Given the susceptibility of FDFT1 transcripts to 8-oxoG modification by air pollution, this study provides support of a relevant physiological role of RNA oxidation in complex metabolic processes that could drive pulmonary dysfunction caused by exposure to air pollution.

## Conclusion

In this study, we demonstrated that the formation of an epitranscriptome mark, such as 8-oxoG, is stimulated by oxidative challenges in air pollution exposures; 8-oxoG accumulation has an adverse effect when accumulated in bronchial epithelial BEAS-2B cells, leading to changes within the cholesterol pathway that result in distinct cellular alterations associated with respiratory health conditions. Our findings also indicate the potential discovery of a new transcriptional biomarker of air pollution effects (FDFT1); a second transcript (DHCR24) will also be better studied in future work as this also represents a potential consistent biomarker of air pollution effect.

Cells exposed to air pollution experienced increased RNA oxidation as compared with clean air controls. Remarkably, higher oxidative air pollution exposures lead to more severely oxidized RNA. Combining direct cell exposure and the 8-oxoG RIP-seq shows that RNA oxidation by air pollution is highly selective as 42 transcripts are consistently oxidized. Our model suggests that induced 8-oxoG marks in mRNA transcripts can affect multifunctional metabolic pathways that are central regulators of cell signaling, proliferation, and survival as well as of maintenance of the structural components. Specifically, the steroid synthesis pathway is enriched in oxidized transcripts. We expect that similar changes in regulatory RNAs (i.e., miRNAs lncRNA, etc.) will have similar consequences to cell function, although these are not examined in this study.

**Fig. 5:**
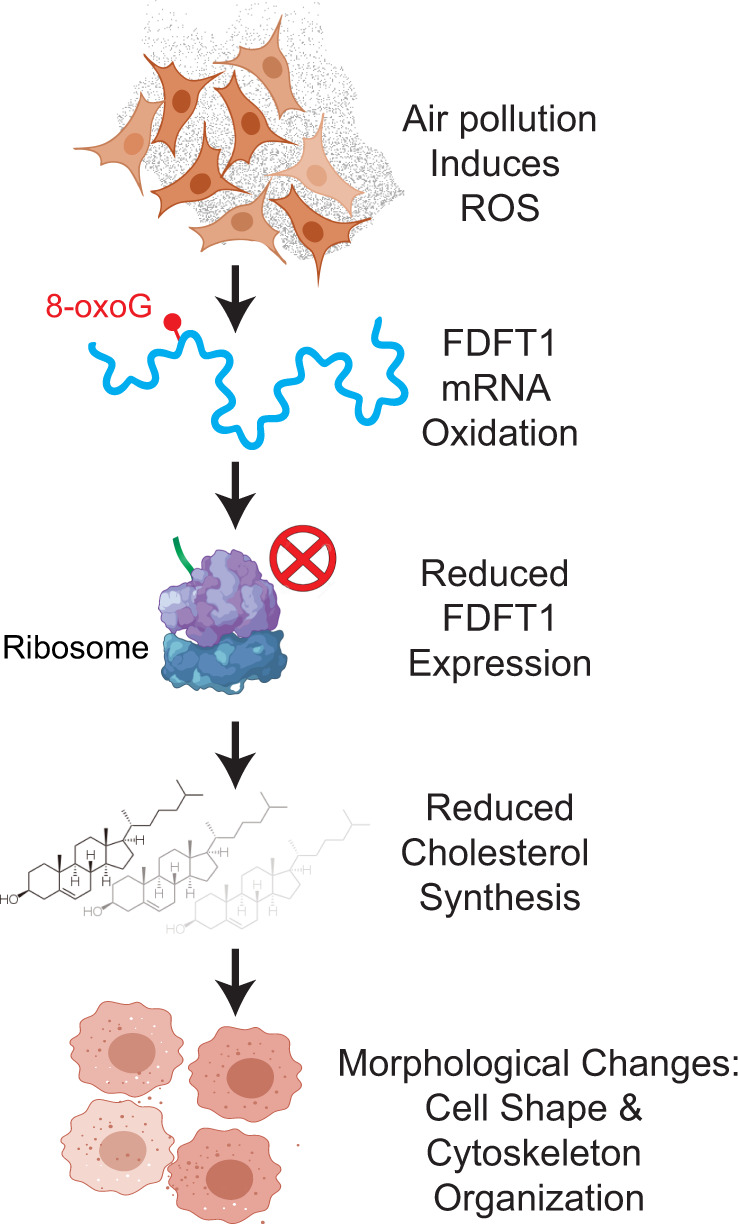
A model of the induction of morphological changes by RNA oxidation in epithelial lung cells exposed to air pollution. Cells exposed to air pollution experienced increased RNA oxidation as compared with clean air controls. RNA oxidation by air pollution is highly selective as 42 transcripts are consistently oxidized. One of these, FDFT1 is highly enriched in 8-oxoG and significantly downregulated at higher oxidative exposures. This event results in reduced FDFT1 protein levels and lower concentrations of intracellular cholesterol. The FDFT1 knockdown promotes cell morphology changes, including transformation of cell shape and reduction of the cytoskeleton organization. These results provide a model linking FDFT1-mediated cholesterol dysregulation and defects in cellular morphology that emerge post exposure to air pollutants.

Most importantly, our results offer, to our knowledge, insights into a previously unknown molecular model of impaired cholesterol biosynthesis that results from aberrantly oxidized FDFT1 transcript by air pollution. The non-mediated decay FDFT1 transcript (FDFT1–215) is highly enriched in 8-oxoG and significantly downregulated at higher oxidative exposures. This event leads to reduced FDFT1 protein levels and lower concentrations of intracellular cholesterol. Subsequently, the knockdown of FDFT1 transforms cell morphology and reduces cytoskeleton organization without affecting cell viability, providing a strong link between FDFT1 dysregulation and defects in cellular morphology that emerge post exposure to air pollutants. Based on these results, we propose the model shown in Fig. 5.

This study has the potential to collectively impact the fields of RNA biology and environmental toxicology. The discovery of 42 consistently oxidized transcripts, including FDFT1 and DHCR24, under different air pollution conditions, could result in the identification of relevant biomarkers for environmental risk assessment. In addition, this data supports the role of FDFT1 RNA oxidation and cholesterol metabolism in normal pulmonary function that could be compromised by air pollution exposures, revealing an exciting mechanism relevant to environmental health. The findings of air pollution reprogramming of cholesterol metabolism have valuable implications in how air pollution may influence lifestyle factors, for example increasing the risk of obesity. Furthermore, the therapeutic relevance of FDFT1 is clear in cases of profound birth defects linked to deficient cholesterol synthesis by recessive variants in FDFT1 transcripts^79^ and FDFT1 regulation may be key for therapeutic intervention of invasive lung cancer cells^92^. Collectively, our observations also open new avenues for RNA biology studies in environmental health science, which may facilitate a better understanding of the principles underlying RNA post-transcriptional modifications and may deepen our knowledge of how molecular changes induced by environmental factors lead to alterations in human physiology.

## Methods

### BEAS-2B cell cultures

BEAS-2B (ATCC CRL-9609) cells were acquired from ATCC. Cell cultures for exposures were initiated from cryopreserved cells (passage 2 from parent stock provided by ATCC) in pre-coated T-75 culture flask following the ATCC instructions. Cells were cultured in 23 ml of complete bronchial epithelial cell growth medium (BEGM, Lonza) with a seeding density of 225,000 cells at 37 °C under an atmosphere containing 5% CO_2_ and in a humidified incubator. Cell were incubated for 4 days until reaching 70–80% confluence with medium renewal every 48 h. Then, cells were passaged to collagen-coated inserts (30 mm diameter, hydrophilic PTFE with pore size of 0.4 µm, EMD Millipore) housed in six-well plates (Corning Costar Clear Multiple Well Plates) with a seeding density of 200,000 cells and incubated for 24 h with 0.8 ml and 1.1 ml of medium in the apical and basolateral side, respectively. Cell culture inserts were coated with 1 ml of 57 µg ml^−1^ of Bovine Collagen Type I (Advanced BioMatrix) in BEGM at least 24 h before seeding. Two hours before exposure, the medium from the apical cell surface was completely removed, and the medium from the basolateral cell surface was renewed with fresh complete medium. Cell density was estimated using 0.6 ml of cell suspension in a Vi-Cell XR viability analyzer (Beckman Coulter).

### Generation of air pollution mixtures

Acrolein (ACR, 90% stabilized, Sigma-Aldrich), methacrolein (MACR, 95% stabilized, Sigma-Aldrich), α-pinene (98% stabilized, Sigma-Aldrich), and O_3_ were mixed inside a 10-m^3^ Teflon chamber at 1 atm, 37.3 °C and with relative humidity (RH) between 35 and 60%, in the dark to generate gas- and particle-phase pollutants. The precise concentrations of acrolein, methacrolein, α-pinene, and O_3_ used in this study are shown in Table 1. Prior to each experiment, a “blank” experiment was performed to test the cleanliness of the chamber and react away residual organics remaining from the previous experiments. The products were then removed by flushing the chamber with dried clean air (<10 particles cm^−3^ and <5 ppb gas-phase impurities) for at least 12 h. Afterward, humidified clean air was flushed through the chamber to raise the relative humidity. On the day of the experiment, acrolein was first injected into the chamber followed by methacrolein, α-pinene, and finally O_3_ at the corresponding concentrations for each exposure level. A set of three experiments were performed under similar initial conditions as shown in Table 1. The O_3_ used for VOC oxidation was produced using an O_3_ generator (TG-10, Ozone Solutions) using UHP O_2_ via corona discharge. Once mixed, these chemicals oxidized and reacted to form gas and particulate phase products. Cell exposure was started after ~45 min of O_3_ injection.

### Physicochemical characterization of air pollution mixtures

Particle size distributions were characterized using a scanning electrical mobility system (SEMS, Brechtel model 2002). The SEMS consists of a differential mobility analyzer (DMA) and a butanol condensation particle counter (CPC). The DMA separates particles based on their electrical mobility, which is a function of the particle diameter. Size-selected particles are counted by the CPC via light scattering. The SEMS is configured to characterize the distribution of suspended particles using 60 discrete size bins ranging from 10 to 1000 nm in diameter, with sheath and polydisperse flow rates set to 5 and 0.35 LPM. A pre-impactor, a NafionTM membrane dryer, and a 210Po strip neutralizer were used to condition the polydisperse sample flow upstream of the DMA column.

The particle-phase bulk chemical composition was measured using an aerosol chemical speciation monitor (ACSM, Aerodyne). Using electron impact ionization, the ACSM can measure the submicron, non-refractory aerosol bulk composition at one minute intervals^44^. Using a standard fragmentation table, the ACSM can speciate the aerosol content into organics, nitrate, sulfate, ammonium, and chloride^44^. The ACSM was calibrated with 300-nm size-selected ammonium nitrate and ammonium sulfate aerosol generated from nebulized 0.005 M solutions to determine the necessary ion-to-mass signal conversion factors using default procedures recommended by the instrument manufacturer. ACSM data were analyzed in Igor Pro V6.37 (Wavemetrics) using ACSM local v1603 (Aerodyne) and other custom routines. Time-dependent air beam corrections were applied to the raw data based on N_2_ signal changes relative to the reference N_2_ signal (when the calibration was performed). The default relative ion transmission efficiency curve was applied to the data. A collection efficiency of 0.5 was assumed for the ACSM, which is consistent with other aerosol mass spectrometers using similar sample inlet and ion generation methods^44^.

A high-resolution time-of-flight chemical ionization mass spectrometer (CIMS, Aerodyne) was used to monitor the molecular composition of gas-phase compounds using (H_2_O)_0–2_H_3_O^+^ clusters as the chemical ionization reagents^93^, with (H_2_O)H_3_O^+^ being the most abundant reagent ion. The chemical ionization used in CIMS is softer than electron impact ionization used in ACSM and can provide information about the molecular composition of gas-phase species. Ionization by (H_2_O)_0–2_H_3_O^+^ clusters proceeds via either the proton transfer, Eq. (1), or the adduct formation, Eq. (2) pathway.

1 $${\mathrm{R}} + \left( {{\mathrm{H}}_{\mathrm{2O}}} \right)_n{\mathrm{H}}_{\mathrm{3O}}^ + \to {\mathrm{RH}}^ + + \left( {n + 1} \right){\mathrm{H}}_{\mathrm{2O}},$$

2 $${\mathrm{R}} + \left( {{\mathrm{H}}_{\mathrm{2O}}} \right)_n{\mathrm{H}}_{\mathrm{3O}}^ + \to R\left( {{\mathrm{H}}_{\mathrm{2O}}} \right)_m{\mathrm{H}}_{\mathrm{3O}}^ + + \left( {n - m} \right){\mathrm{H}}_{\mathrm{2O}}.$$

The sensitivity of the CIMS (e.g., conversion ion intensity of RH^+^ to mass concentration for R) depends on the proton affinity of the analyte R, the abundance of the reagent ions (i.e., amount of (H_2_O)_0–2_H_3_O^+^ available, the relative distribution of which varies with sample gas humidity as well), and other instrument factors (e.g., reaction timescale between reagent ion and analyte; ion-transmission efficiencies) and requires calibration with authentic standards, which are not commercially available or practically viable for the hundreds and possibly more oxidation products observed.

### Air–liquid interface (ALI) exposures of BEAS-2B cells

BEAS-2B cells were exposed for 1.5 h to low concentrations of VOCs + O_3_ precursors (97 ppb methacrolein, 100 ppb acrolein, 44 ppb α-pinene, and 109 ppb O_3_) and high concentrations of VOCs + O_3_ precursors (670 ppb methacrolein, 790 ppb acrolein, 0 ppb α-pinene, and 3900 ppb O_3_) as shown in Table 1. Cells were exposed in two polycarbonate modular cell exposure chambers (MIC-101 Billups-Rothenberg), used to house exposed and control samples. Prior to each exposure, the modular chambers were conditioned with O_3_ flush to reduce contamination by plasticizer residues (which were initially found to be responsible for O_3_ loss), followed by clean air flush to displace residual O_3_. Probes (HMP60) were used to monitor the RH and temperature downstream from each chamber. Each chamber held two or three six-well plates, and a mix of 0.08 LPM CO_2_ (UHP, Airgas) and 1.52 LPM air pollutants was pumped through the exposure chamber for 1.5 h. In parallel, a mix of 0.08 LPM CO_2_ and 1.52 LPM humidified clean air was pumped through the control chamber. The modular exposure chambers were housed in a temperature-controlled room at 37 °C.

### RNA extractions

Following exposure, each membrane was treated with 1 ml of TRIzol (Invitrogen) in the apical side and gently mixed to ensure thorough lysis. The whole lysate was collected and frozen until the day of the extraction. TRIzol RNA extraction was conducted following TRIzol’s manufacturer instructions. To prevent artificial oxidation of RNA by dissolved oxygen in solutions, ethanol (200 Proof, OmniPur, EMD Millipore), isopropanol (molecular biology grade, IBI Scientific), and nuclease-free water (Ambion) used in the downstream steps after TRIzol were purged with ultra-high purity N_2_ for 30 min. TRIzol aliquots were thawed on ice, and RNA was purified using Direct-zol RNA miniprep (Zymo Research). The purified RNA was incubated with DNAse I (NEB) following the manufacturer’s protocol, and then re-extracted with RNA clean and concentrator kit (Zymo Research).

### Ex vivo exposure of RNA to air pollution

We extracted total RNA from BEAS-2B cells as described above and stored at −80 °C. The day of the exposure, 8 µg of RNA were resuspended in 500 µl of TE buffer (pH. 8.0) supplemented with 10 µl of SUPERaseIn RNA inhibitor (Invitrogen) into each well of a six-well plate. The ex vivo exposures were conducted using the high-level air pollution mixture of the VOC + O_3_ precursors (Table 1) for 1.5 h following the same conditions as for the BEAS-2B exposures. After exposure, RNA was purified with RNA clean and concentrator kit (Zymo Research) and then stored at −80 °C until the day of analysis.

### Quantification of free 8-oxoG levels in the total RNA

Free 8-oxoG was quantified in the total RNA using the DNA/RNA Oxidative Damage ELISA Kit (Cayman Chemical). Two RNA dilutions (3 µg and 1.5 µg of the total RNA) were digested with 0.375 µg of nuclease P1 from *Penicillium citrinum* (Sigma-Aldrich) in 20 mM sodium acetate buffer pH 5.2 containing 50 mM sodium chloride and 0.1 mM zinc chloride in a 105 µl of reaction volume. After incubation at 37 °C for 2 h, 1 unit of calf intestinal phosphatase (CIP, NEB) and 5× alkaline phosphatase buffer (500 mM Tris acetate, 220 mM sodium chloride, 50 mM magnesium chloride, pH 7.9) was added to a final reaction volume of 150 µl. The competitive ELISA method was conducted at the two dilutions (1 µg and 0.5 µg of the total RNA) with three technical replicates following the steps in the manufacturer protocol. The standard curve, measured as B/B_0_ (standard bound/maximum bound) for each standard dilution, was calculated from triplicate standard readings. The sample concentration was determined in the linear range of the standard curve (10.3–3000 pg ml^−1^), after accounting for the dilution, with a sensitivity (determined as 80% B/B_0_) of 10.3–11.8 pg ml^−1^ and a mid-point (defined as 50% B/B_0_) of 52–104 pg ml^−1^. A disparity lower than 20% between the different dilutions was considered acceptable. In addition, we corrected the cross-reactivity of the antibody for 8-oxoG in RNA using a factor of 0.38 as suggested in the manufacturer protocol. Buffers were prepared fresh on the day of the assay using N_2_-purged nuclease-free water.

### 8-oxoG RIP-seq analysis

Immunoprecipitation of 8-oxoG-containing RNA was performed in two biological replicates for each condition. After DNase I treatment of RNA, ribosomal RNA (rRNA) was depleted using Ribo-Zero Gold rRNA Removal kit (Illumina) as described by the manufacturer. Depletion of rRNA was validated by Agilent 2100 Bioanalyzer (Agilent), and all samples had a RIN higher than 7. All buffers were prepared fresh from concentrated stocks on the day of pulldown experiments using N_2_-purged nuclease-free water. RNA was incubated with 12.5 µg of 8-oxo-7,8-dihydroguanine (8-oxoG) monoclonal antibody (0.5 mg ml^−1^, Clone 15A3, Trevigen) in IP buffer (10 mM Tris pH 7.4, 150 mM NaCl, 0.1% IGEPAL, and 200 units ml^−1^ of SUPERaseIn RNA inhibitor (Invitrogen) in a 1 ml of reaction volume for 2 h on a rotator at 4 °C. Then, SureBeads Protein A magnetic beads (Biorad) were washed according to the manufacturer’s recommendation and blocked in IP buffer supplemented with 0.5 mg ml^−1^ bovine serum albumen (BSA) for 2 h at room temperature. After washing beads twice in IP buffer, they were resuspended in IP buffer, mixed with the RNA–antibody reaction and then incubated for 2 h on a rotator at 4 °C. Next, the beads were washed three times in IP buffer before performing two competitive elutions with free 8-oxodG nucleosides (Cayman Chemical). Each elution was conducted by incubating the beads with 108 µg of 8-oxodG in IP buffer for 1 h on a rotator at 4 °C. Then, the elution volume was cleaned up using the RNA Clean and Concentrator-5 kit (Zymo Research).

Input RNA and immunoprecipitated 8-oxoG-containing RNA libraries were prepared using the NEBNext Small RNA kit (NEB) by the Genomic Sequencing and Analysis Facility at the University of Texas at Austin. For the samples generated at low air pollution levels, sequencing was performed on an Illumina NextSeq 500 paired-end 2 × 75 bases with a read depth of 20 M reads for pulldowns and 32 M reads for input RNA samples. For the samples generated at high air pollution levels, sequencing was performed on an Illumina HiSeq 4000 pair-end 2 × 150 bases with a with a read depth of 16 M reads for pulldowns and 32 M reads for input RNA samples.

### Transcriptomics analysis

FastQC was used to generate quality check reports on the raw data, and then read trimming was performed using cutadapt 1.14, followed by another quality check using FastQC that demonstrated high-quality read data. These preprocessed data were then aligned to the Ensembl comprehensive human genome annotation (GENCODE 26, GRCh38.p12) using STAR 2.6.0c, allowing novel splice junctions and using a two-pass mapping approach (transcriptome reference assembly then realignment to the reference) for comprehensive transcriptome alignment. Alignment was performed using parameters recommended in the STAR manual for ENCODE standards with a resultant mapping rate of >60% for all samples and multi-mapping rates of 9–32%. Next, RSEM 1.3.1 was used to estimate transcript abundances, and then differential expression and 8-oxoG enrichment analysis were performed using DESeq2 in R version 3.6.1. Transcripts were annotated using biomaRt in R. The analyzed datasets are available in Supplementary Data 1–15.

### Enrichment analysis

Enrichment analysis of the differentially upregulated, downregulated, and oxidized transcript lists was performed in Enrichr web tool^71^. We generated the lists for enrichment by filtering the transcripts with adjusted *P*-value < 0.05, and fold change >2 (for upregulated genes) and <0.5 (for downregulated genes). The list of oxidized transcripts was obtained for enriched genes (positive fold change) and with an adjusted *P*-value < 0.05 for high air pollution levels and adjusted *P*-value < 0.1 for low air pollution levels. The plots of the top-most enriched pathways were generated from the KEEG database by ranking them by the Enrichr’s combined score^71^. The pathway enrichment results for the high-level and low-level exposures transcriptomics as well as high-level and low-level exposures 8-oxoG enrichment are available in Supplementary Data 4, 8, 9, 10, 14, 15.

### Validation of 8-oxoG immunoprecipitation

All the buffers were prepared fresh on the day of the assay using N_2_-purged nuclease-free water to prevent artefactual oxidation. A 24-mer 8-oxoG RNA oligonucleotide (with sequence: [NN(8-oxoG)N]_6_, where N is A, G, C, or U) and the 24-mer unmodified RNA oligo (with sequence: [NNGN]_6_) were custom synthesized by GeneLink. The oligos were radiolabeled using T4 polynucleotide kinase (NEB), as described by the manufacturer. After labeling, RNA was cleaned up by ethanol precipitation. This was done by first adding 1 M Tris buffer (pH 8.0) and 1 M sodium acetate (pH 5.2) to the reaction mixture to bring the final concentrations to 50 mM and 0.3 M, respectively. Two volumes of phenol/chloroform/isoamyl alcohol (25:24:1) (Fisher Scientific) were then added, and the solution was vortexed for 1 min followed by centrifugation at 15,000 *g* for 2 min to achieve phase separation. The aqueous (top) phase was collected, and 1 µl of GlycoBlue Coprecipitant (Thermo Fisher) and 2.5 volumes of chilled 100% absolute ethanol (OmniPur, 200 Proof, Millipore Sigma) were added. The solution was mixed and then incubated overnight at −20 °C. The following day, the solution was centrifuged at 4 °C at 15,000 *g* for 15 min. The supernatant was removed, and then washed with 95% ethanol followed by a final centrifugation at 15,000 *g* for 5 min. The supernatant was discarded, and the pellet was dried in a Vacufuge plus (Eppendorf) for 5 min before resuspension in Molecular Biology Grade Water (Quality Biological).

To generate the input RNA for 8-oxoG IP, 2.5 ng of the P^32^-labeled RNA (either 8-oxoG or unmodified) was mixed with 5.1 µg of unmodified oligomer and resuspended in 56 µl of N_2_-purged Molecular Biology Grade Water (Quality Biological). The 8-oxoG immunoprecipitation was conducted as described above. After elution, the P^32^ signal was detected using liquid scintillation counter (Beckman LS 6500).

### Dot blot assay

All RNA oligomers used to test the specificity of the commercially available 8-oxoG antibody (clone 15A3) employed in 8-oxoG RIP-seq are listed in Supplementary Table 1 and were synthesized by GeneLink. Serial twofold dilutions of each oligo were denatured, and 5 µl was spotted on the hybond-N+ nylon membrane (GE Healthcare) followed by UV-cross-linked at 120,000 μJ cm^−^^1^ for 60 s. The membrane was blocked with 5% bovine serum albumin (BSA; Fisher Scientific) in 1× PBS (pH 7.4, VWR) containing 0.05% Tween 20 (VWR) overnight at 4 °C. After extensive washing, it was incubated at 4 °C in 1% BSA in 1× PBS with the addition of anti-8-oxoG antibody (clone 15A3, Trevigen) used at 1:400 dilution. Following extensive washing, the membrane was incubated at room temperature for 1 h with anti-mouse IgG H&L HRP conjugate (W4021, Promega) secondary antibody diluted 1:2500 in in 1% BSA in 1× PBS. Chemiluminescent detection was conducted on a ChemiDoc XRS+ imaging system (Biorad) and quantification of the band’s intensity with CLIQS (TotalLab).

### Reverse transcription truncation assay

All the buffers were prepared fresh on the day of the assay using N_2_-purged nuclease-free water to prevent artefactual oxidation. Chemical labeling of RNA was conducted by mixing 1 µg of the total RNA extracted from BEAS-2B cells after exposure with 100 μl of 100 mM NaPi buffer (pH 8.0) (Sigma Aldridge), 5 μl of 500 mM BTN-NH2 (EZ-Link Amine-PEG2-Biotin; Thermo Fisher Scientific) and 1 μl of SUPERase In RNase inhibitor (Thermo Fisher Scientific) following by incubation at room temperature for 10 min. Next, 6.3 μl 100 mM K_2_IrBr_6_ (Alfa Aesar) was added and allowed to react for 30 min at room temperature. The reaction was quenched with 4 μl of 20 mM EDTA solution at pH 8.0 (Thermo Fisher Scientific). The RNA was purified with the RNA Clean and Concentrator-5 kit (Zymo Research) before reverse transcription.

cDNA products from FDFT1–215, GAPDH, and PPIB RNA were synthesized with SuperScript IV Reverse Transcriptase (Thermo Fisher Scientific) using primers listed in Supplementary Table 2. We followed the steps suggested by the manufacturer. Briefly, 2.2 µg of RNA was annealed with 2 µM of each primer and 10 mM dNTP mix for 5 min at 65 °C, and then incubated on ice for at least 1 min. Then, the following components were added: 5× SSIV Buffer, 100 mM DTT, SuperScript IV Reverse Transcriptase, and SUPERase In RNase inhibitor. The mixture was incubated at 55 °C for 10 min and then at 80 °C for 10 min to terminate the reaction. To remove RNA templates, the cDNA products were incubated with two units of RNase H (NEB) at 37 °C for 15 min.

PCR was carried out with the pairs of primers listed in Supplementary Table 2. We combined 2 µl of cDNA product with primers (final concentration of 300 nM of each primer) and 1× Power Sybr Green PCR Master Mix (Thermo Fisher Scientific) in a final reaction of 50 µl. The reactions started at 95 °C for 10 min and cycled 40 times at 95 °C for 15 s and 60 °C for 1 min. PCR products were resolved on a 3% agarose gel with DNA size markers and stained with ethidium bromide. Bands were detected on a ChemiDoc XRS+ imaging system (Biorad) and quantification of the band’s intensity with CLIQS (TotalLab). The full, uncropped gel images are shown in Supplementary Fig. 14.

### Cytotoxicity analysis

Cell viability was measured by trypan blue exclusion assay. Before the assay, cells were rinsed with warmed phosphate buffer solution pH 7.4 (PBS, Thermo Fisher Scientific) and then trypsinized with 0.5% polyvinylpyrrolidone (Sigma-Aldrich) in trypsin/EDTA 0.025% solution (Lonza) for 6 min at 37 °C. Then, trypsin-neutralizing solution (Lonza) was added following by centrifugation at 130 rpm for 5 min. The cell pellet was resuspended in 5 ml of fresh cell media. Cell viability was estimated using 0.6 ml of cell suspension in a Vi-Cell XR viability analyzer (Beckman Coulter).

Cellular membrane damage was measured by detection of lactase dehydrogenase (LDH) in the cellular medium using a colorimetric assay (LDH Cytotoxicity Detection Kit, Takara Bio). Absorbance of the assay was measured at 491 nm for 30 min at 25 °C using a Cytation 3 plate reader with constant shacking (Biotek).

### Western blotting and cholesterol analysis

Cells attached to the cell culture inserts were lysed by adding 200 μl of M-PER mammalian protein lysis buffer (Thermo Fisher Scientific) supplemented with Halt protease inhibitor cocktail (Thermo Fisher Scientific) with vigorous mixing by pipetting. The lysate was stored at −80 °C until the day of analysis, and protein concentrations were analyzed by Coomassie (Bradford) protein assay kit (Thermo Fisher Scientific). The whole protein lysate was dissolved in 5% 2-mercaptoethanol sample buffer (3× buffer: 0.5 M Tris-HCl pH 6.8, 10% (w/v) SDS, 25% glycerol, and 0.5% (w/v) bromophenol blue). Electrophoresis of 0.5–5 μg of protein loaded per lane was conducted in 10% polyacrylamide gels at 90 V for 2.5 h. Protein bands in the gel were transferred to 0.2-µm nitrocellulose membranes (Biorad) using a Trans-Blot SD Semi-Dry Transfer Cell (Biorad). Then, membranes were blocked overnight in 5% skimmed milk in Tris-buffered saline containing 0.05% Tween 20 (VWR). Squalene synthase (FDFT1) was detected with rabbit monoclonal anti-FDFT1 IgG [EPR16481] (ab195046, Abcam) used at 1:5000 dilution, and goat anti-rabbit IgG H&L HRP conjugate (ab6721, Abcam) was used as a secondary antibody at 1:10,000 dilution. Immunodetection was performed with the Clarity Western ECL substrate (Biorad). Prior to detection of GAPDH as loading control, the membrane was stripped with mild stripping buffer (200 mM glycine, 0.1% (w/v) SDS, and 1% Tween 20). Then, the membrane was blocked and reblotted using mouse monoclonal GAPDH Antibody [6C5] (Santa Cruz Biotechnology). Polyclonal anti-mouse IgG H&L HRP conjugate (Promega) was used as secondary antibody. Chemiluminescent detection was conducted on a ChemiDoc XRS+ imaging system (Biorad) and quantification of the band’s intensity with CLIQS (TotalLab). The full, uncropped western blot images are shown in Supplementary Figs. 15, 16.

Intracellular cholesterol was quantified in whole cellular lysates using the Amplex Red Cholesterol Assay kit (Thermo Fisher Scientific) according to the manufacturer instructions. Cholesterol was measured in two biological replicates, and each sample was quantified in triplicate.

### Confocal microscopy

Prior to fixing of the cells, membranes were removed from the plastic insert by making an incision around the edge of the membrane. Each membrane was then placed onto a microscope slide mounted in a Petri dish with cells facing upward. Cells were fixed in 1 ml of 3.7% formaldehyde solution in phosphate buffer solution pH 7.4 (PBS, Thermo Fisher Scientific) for 15 min at 37 °C. After fixation, the formaldehyde solution was discarded, and the membrane was washed three times with 1 ml of PBS pre-warmed to 37 °C. Then, 1 ml of 0.1% Triton-X-100 (Sigma-Aldrich) in PBS was placed onto the membrane for 4 min and washed with 1 ml of PBS three times. The membrane was then pre-incubated with 1 ml of 1% bovine serum albumin (BSA) in PBS for 20 min, prior to adding the phallotoxin staining solution. To stain F-actin in the cells, 10 µl of Alexa Fluor 594 Phalloidin solution (Thermo Fisher Scientific) was diluted into 400 µL of PBS with 1% BSA solution. The staining solution was placed onto the membrane for 20 min at room temperature and protected from light to prevent photobleaching. The fluorescent media was aspirated and washed three times with PBS. Once each membrane was stained, a drop of ProLong Gold Antifade Mountant with DAPI (Thermo Fisher Scientific) was placed onto the membrane. A coverslip was positioned on top of the membrane, and then the edges of each coverslip were sealed with clear nail polish and left to dry. Specimens were stored in the dark at 4 °C until the day of analysis. Confocal microscopy for analysis was performed using a Zeiss LSM 710 Confocal Microscope. Five or more images were acquired in random locations and captured using Zen Pro software with a ×63 oil objective and filters for DAPI and Alexa 594.

### Image analysis

The extent of F-actin area was quantified in Fiji/ImageJ by drawing the outline of the cell with the free hand pencil tool in at least five cells in three confocal images (×63 optical magnification) selected for each biological replicate and condition. The F-actin organization around the nucleus and plasma membrane was quantified using Fibriltool plug-in in Fiji according to the described protocol^87^. This analysis was conducted in three confocal images (×63 optical magnification) selected for each biological replicate and condition. The anisotropic score was computed on five or more cells per image by drawing an area of interest of ~5 µm by 10 µm.

### Knockdown of FDFT1 in BEAS-2B cells

BEAS-2B cells were cultured on collagen-coated inserts as described above with a seeding density of 225,000 cells 24 h before transfection. To knock down FDFT1, we used a predesigned siRNA (s138, Silencer Select, Thermo Fisher Scientific) to target FDFT1 main coding transcripts (si138). In addition, a custom siRNA (si215) was designed to target FDFT1–215 (Transcript ID ENST00000529464.5) with antisense sequence 5′-GCCAACUCUAUGGGCCUGUUU-3′. As negative control, we used the scrambled siRNA Silencer Select Negative Control No. 1 siRNA from Thermo Fisher Scientific.

SiRNAs were transfected using Lipofectamine 3000 Reagent (Thermo Fisher Scientific), according to the manufacturer’s protocol. Briefly, the RNA master mix was prepared by diluting 37.5 pmol of the siRNA in 125 µl of Opti-MEM medium (Thermo Fisher Scientific). Then, the Lipofectamine master mix was prepared by mixing 125 µl of Opti-MEM medium with 3.75 µL of Lipofectamine 3000 following by an incubation at room temperature for 5 min. To prepare the transfection complexes, the lipofectamine master mix was slowly added, dropwise, to the RNA master mix. The solution was then gently mixed and incubated at room temperature for 30 min. During this incubation, the basolateral media was refreshed, and the apical media was completely removed. Following the incubation, the transfection complex was added on the apical side, and then 550 µl of fresh BEGM medium was added dropwise and gently rocked. Cells were incubated at 37 °C for 24 h in a humidified 5% CO_2_ incubator. To establish the transfection efficiency, we transfected cell using BLOCK-IT fluorescent oligo (Thermo Fisher Scientific) and we visualized using a Zeiss Axiovert 200 M Widefield Fluorescent Microscope and a FITC filter. RNA and protein were extracted, and formaldehyde fixation of cells was performed following the protocols described above.

### Statistics and reproducibility

We conducted all described measurements at least twice using independent and biological replicates. The experiments were not randomized, and the researchers were not blinded during experiments. Sample size is indicated in figure labels. All data were presented as the mean ± one standard deviation. Statistical analysis between groups was performed in Microsoft Excel and GraphPad Prism 8 software determined by t test (one-tailed homoscedastic) or Mann–Whitney test.

### Reporting summary

Further information on research design is available in the Nature Research Reporting Summary linked to this article.

## Supplementary information

Supplementary Information

Description of Additional Supplementary Files

Supplementary Data 1–15

Supplementary Figures

Reporting Summary

## Data Availability

All the raw data using RNA sequencing and the analyses generated during the current study are available in NCBI Gene Expression Omnibus (GEO) under accession number GSE137019. Source data underlying the graphs and charts presented in the main figures is provided in the Supplementary Data 16. All other relevant data can be obtained from corresponding author upon reasonable request
