# Supplementary material for: Post-transcriptional air pollution oxidation to the cholesterol biosynthesis pathway promotes pulmonary stress phenotypes
Source: Commun Biol. 2020 Jul 22;3:392. doi: 10.1038/s42003-020-01118-6 (PMC7376215; doi:10.1038/s42003-020-01118-6)
Supplement: Supplementary file 1 — Supplementary Information [file 42003_2020_1118_MOESM1_ESM.pdf]

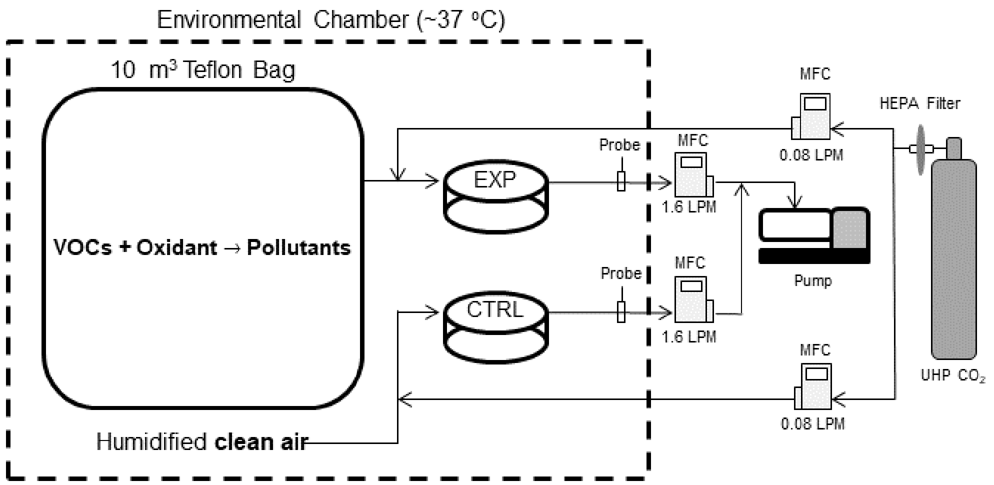


**Supplementary Fig. 1**

Schematic depicting the experimental setup used to expose BEAS-2B cells with air pollution mixtures. We used a temperature-controlled environmental chamber at 1 atm containing a 10 m$^3$ Teflon bag and two cell exposures chambers. Probes were used to monitor the temperature and relative humidity inside each exposure chamber.

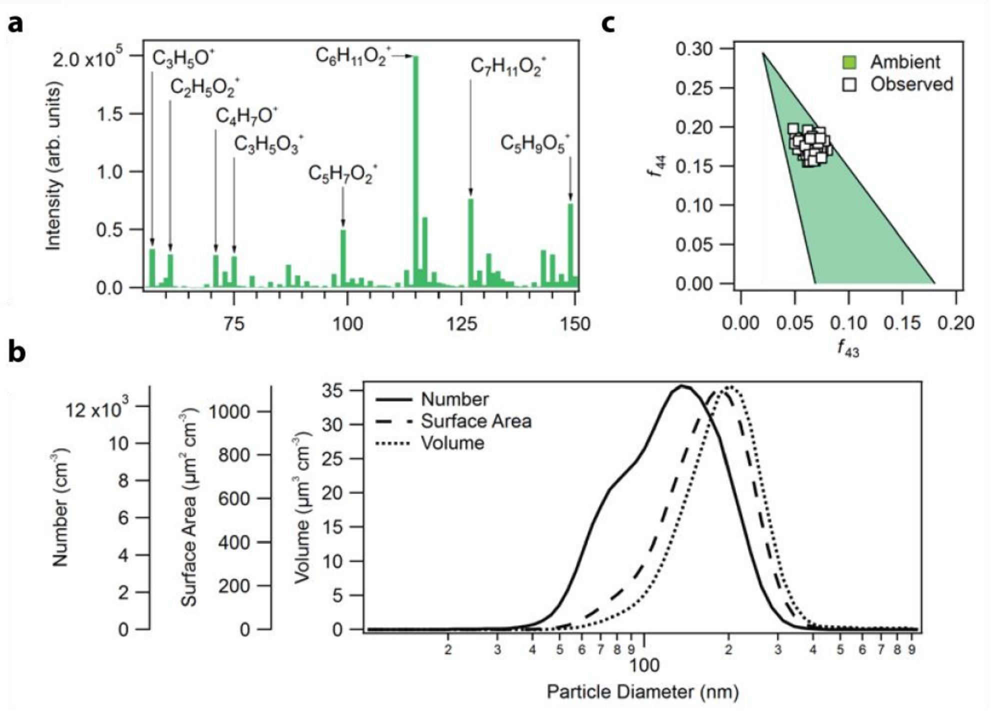


**Supplementary Fig. 2**

Physicochemical characterization of the air pollution mixture with higher oxidative potential. **a** Gas-phase composition observed during the exposure period (0 – 1.5 hour) using the chemical ionization mass spectrometer (CIMS). Average integrated unit-mass ion intensities are shown. Labels indicate select dominant ions observed at the corresponding m/z. Ions ranging between m/z 2-56 and 151-400 were monitored but not shown. Precursor volatile organic compounds are detected as $C_3H_5O^+$ (ACR, $C_3H_4O$) and as $C_4H_7O^+$ (MACR, $C_4H_6O$). The integrated ion intensities shown are not adjusted for sensitivities due to lack of authentic standards for oxidation products. **b** Size distribution of secondary organic aerosol as observed by the scanning electrical mobility system (SEMS), averaged over the period between 0 to 1.5 hour from the start of the exposure. Lognormal distributions are shown. **c** Typical $f_{43}$ vs $f_{44}$ profile, an estimator for aerosol oxidation state, observed by the aerosol chemical speciation monitor during the exposure period (0-1.5 hour).

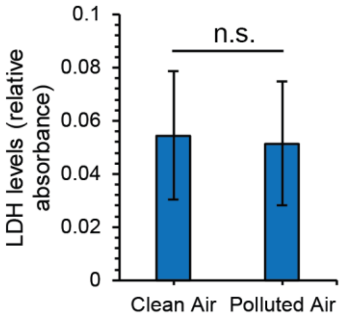


**Supplementary Fig. 3**

LDH levels after exposure of BEAS-2B cells to the air pollution mixture (lower oxidative mixture in Table 1) for 1.5 h. Activity of LDH was measured by a colorimetric assay in the cell culture media (n = 3 independent experiments). Error bars are expressed as one standard deviation (s.d.), n.s. refers to no significance difference was determined by *t-test* (one-tailed homoscedastic) with significance established as p-value < 0.05.

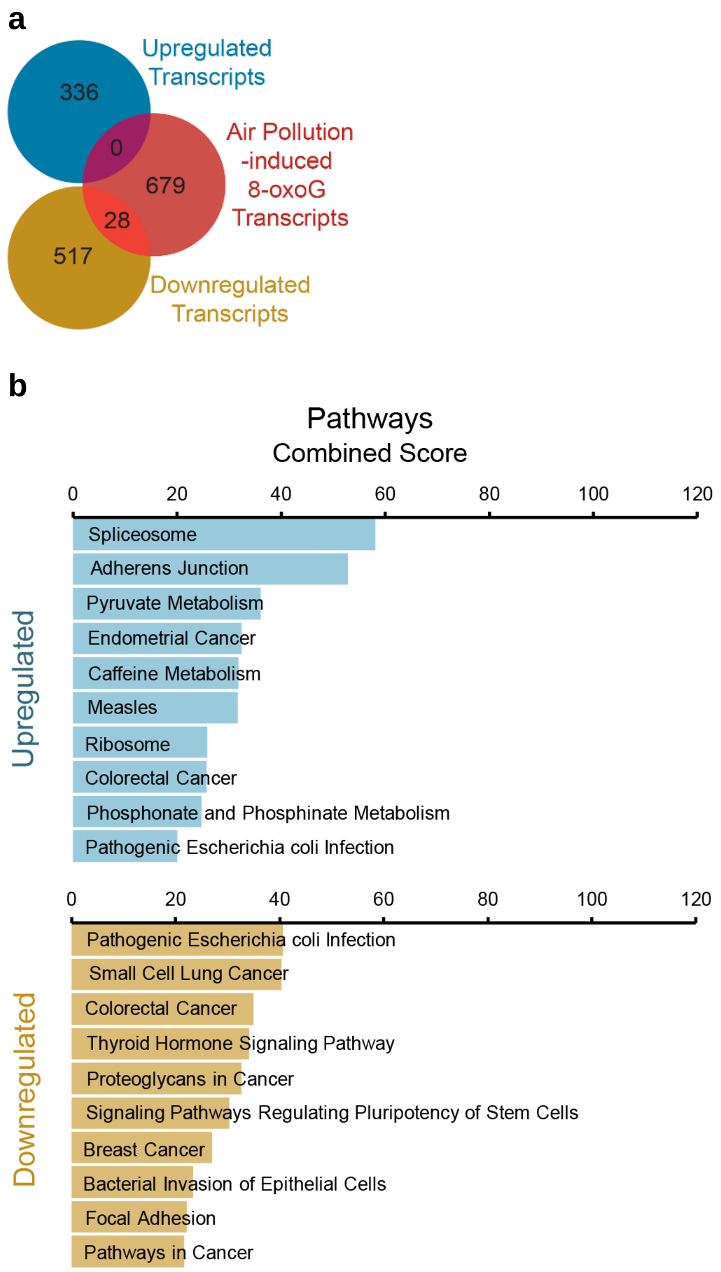


**Supplementary Fig. 4**

**a** Venn diagram shows the number of transcripts upregulated and downregulated in BEAS-2B cells following low-level exposure, and the overlap with oxidized transcripts after exposure. **b** Top ranked KEGG pathways enriched from the differentially expressed transcripts (upregulated and downregulated). We conducted transcriptomics analysis of the mRNAs (e.g., using a fraction of the input mRNA pool) to compare expression changes under exposed and control cells. This analysis shows differential expression of 878 mRNA transcripts with an adjusted p-value $< 0.05$. A lower p-value cutoff was used given the low variance in the transcriptome data as compared to the 8-oxoG pulldowns.

Of these, 336 transcripts exhibit increased expression with a fold change > 2, and 545 exhibit decreased expression with fold change < 0.5 (Supplementary Data 1 and 2).
Terms ranked by the combined score in Enrichr. Genes associated to each pathway are presented in Supplementary Data 9 and 10.

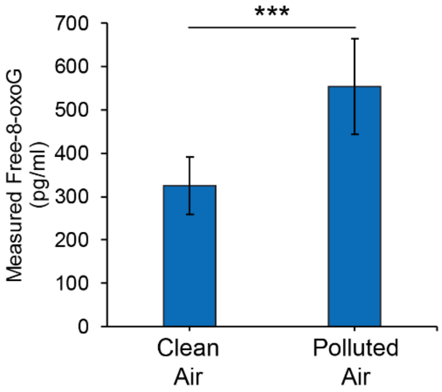


**Supplementary Fig. 5**

Exposure of BEAS-2B cells to the high-level air pollution mixture (Table 1) for 1.5 h. Free 8-oxoG nucleosides from total RNA were quantified shortly after exposure (at t = 1.5 h) by ELISA (n = 3 independent experiments). Statistical difference was computed using *t-test* analysis (one-tailed homoscedastic) and significance is denoted as *** for p-value < 0.0005. Error bars are expressed as one standard deviation (s.d.).

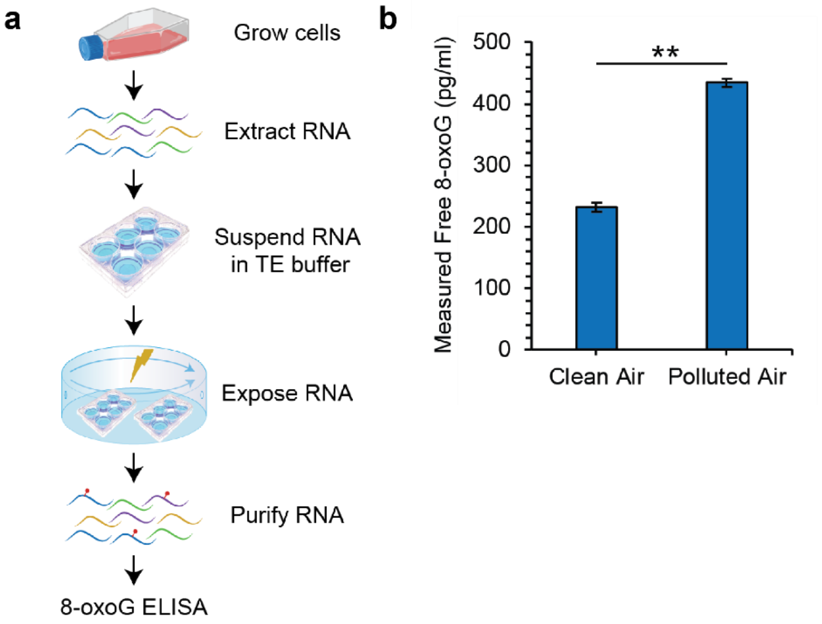


**Supplementary Fig. 6**

Detection of 8-oxoG in *ex vivo* exposure of total RNA to the high-level air pollution mixture. **a** Schematic depicting the direct exposure of RNA to air pollution. Cells were grown until reaching confluence. Cells were lysed with Trizol and then RNA was extracted and purify using spin column-based purification. 8 µg of total RNA was resuspended in 500 µl of TE buffer (pH 8.0) and exposed to air pollution for 90 min (using the higher concentrations of the VOC+$O_3$ precursors in Table 1). RNA was purified and 8-oxoG was measured with ELISA. **b** Quantification of free 8-oxoG nucleosides from total RNA directly exposed to air pollution using ELISA (n = 3 independent experiments). Statistical difference was computed by *t*-test analysis and significance is denoted as ** for p-value < 0.001. Error bars are expressed as one standard deviation (s.d.).

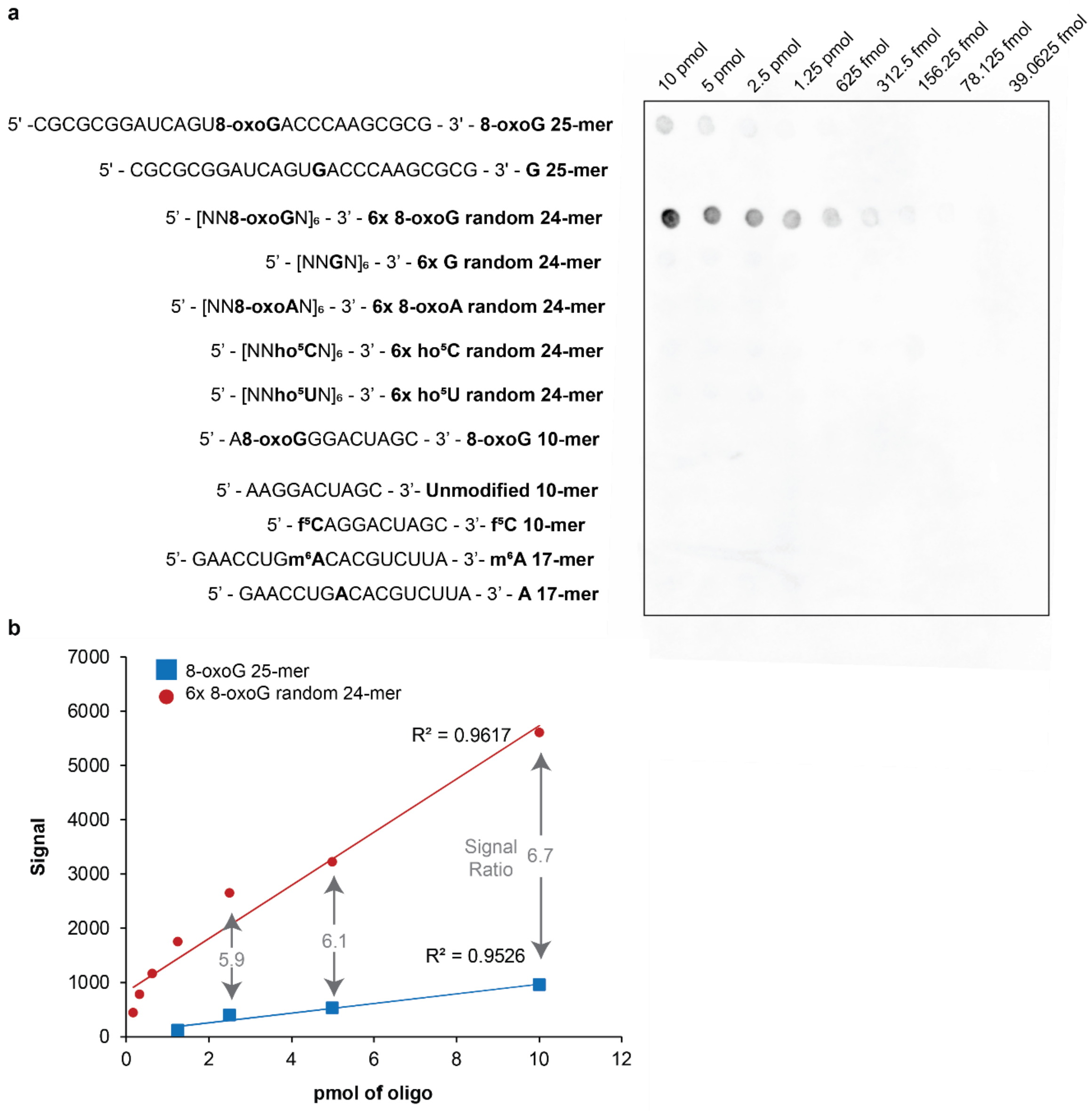


## Supplementary Fig. 7

Assessment of the anti-8-oxoG antibody (clone 15A3) demonstrating high specificity of the antibody. **a** Dot blotting of different RNA oligonucleotides containing common methylated and oxidized RNA modifications as described in the label. Decreasing amounts (indicated on top of the blot) of the oligos were spotted onto the membrane, UV crosslinked and probed with anti-8-oxoG antibodies. **b** Quantification of the signal detected for the 8-oxoG 25-mer with one modification (square) and the 6x 8-oxoG random 24-mer containing six modifications (circle). Quantification of the signal was conducted in CLIQS (TotalLab), with background subtracted. The signal ratio of 6x 8-oxoG random 24-mer/8-oxoG 25-mer of ~6 is proportional to the ratio of modifications in each oligomer at 10, 5 and 2.5 pmol of oligomers.

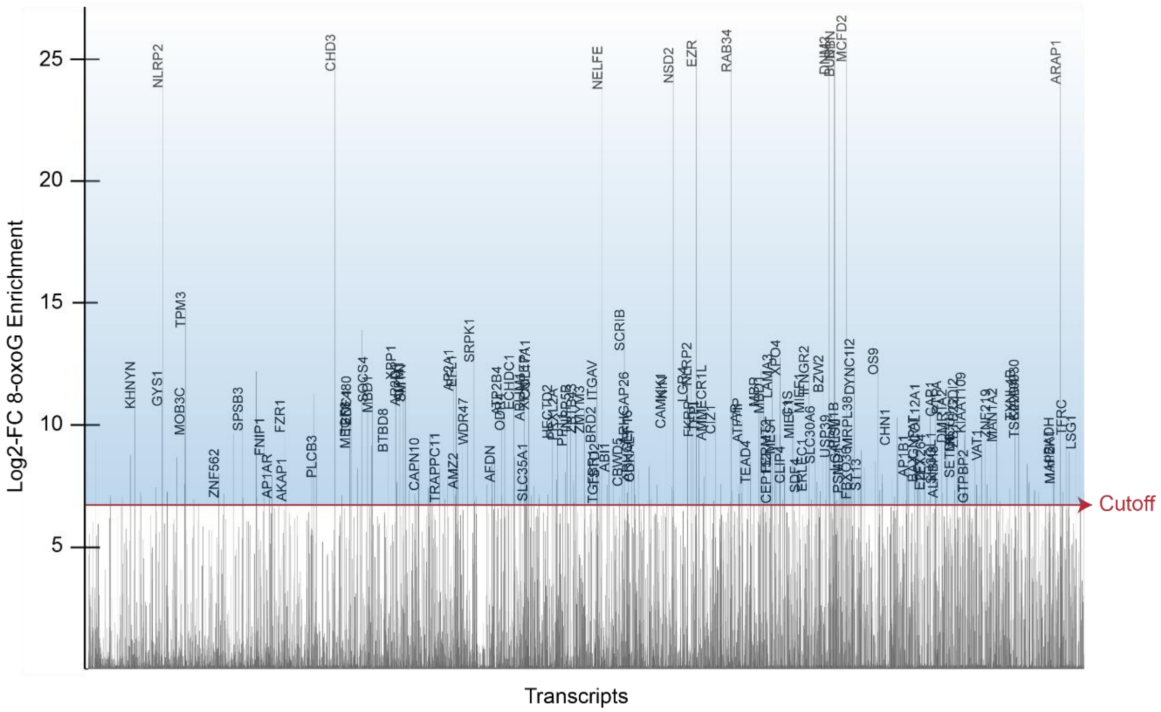


## Supplementary Fig. 8

Log2-FC plot representing selected 20% of all detected 8-oxoG enriched transcript (5493 out of 27,269 transcripts) in exposed cells. Labeled transcripts refer to the ones in the subset of the identified 707 oxidized transcripts by air pollution. This plot demonstrates that after applying the comparisons in Fig. 2D, the minimum log2-FC of 6.7 provides a stringent threshold cutoff for removal of background noise from artifactual oxidation.

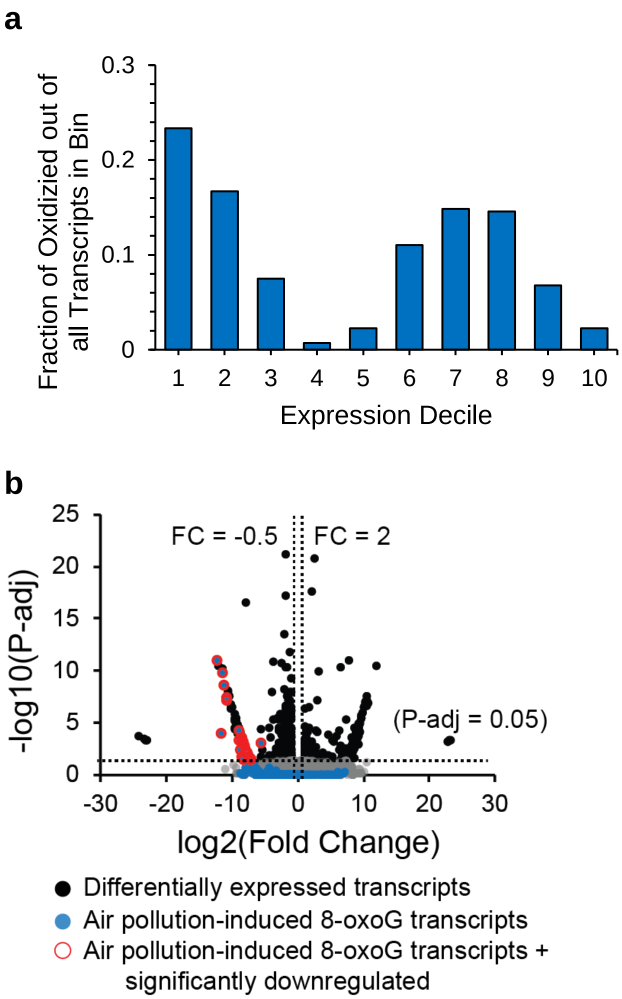


**Supplementary Fig. 9**

**a** Fraction of oxidized transcripts out of all transcripts (within one expression bin) in BEAS-2B cells exposed to low-level air pollution (Table 1). **b** Volcano plot shows differential expression by comparing the input mRNA pool between air pollution vs clean air conditions at the low-level exposure (Table 1). Data points in blue are 8-oxoG enriched and data points in red are 8-oxoG enriched and differentially downregulated (Supplementary Data 3). Significant expression was established with a fold change < 0.5 or > 2 with a statistical confidence of $\alpha = 0.1$.

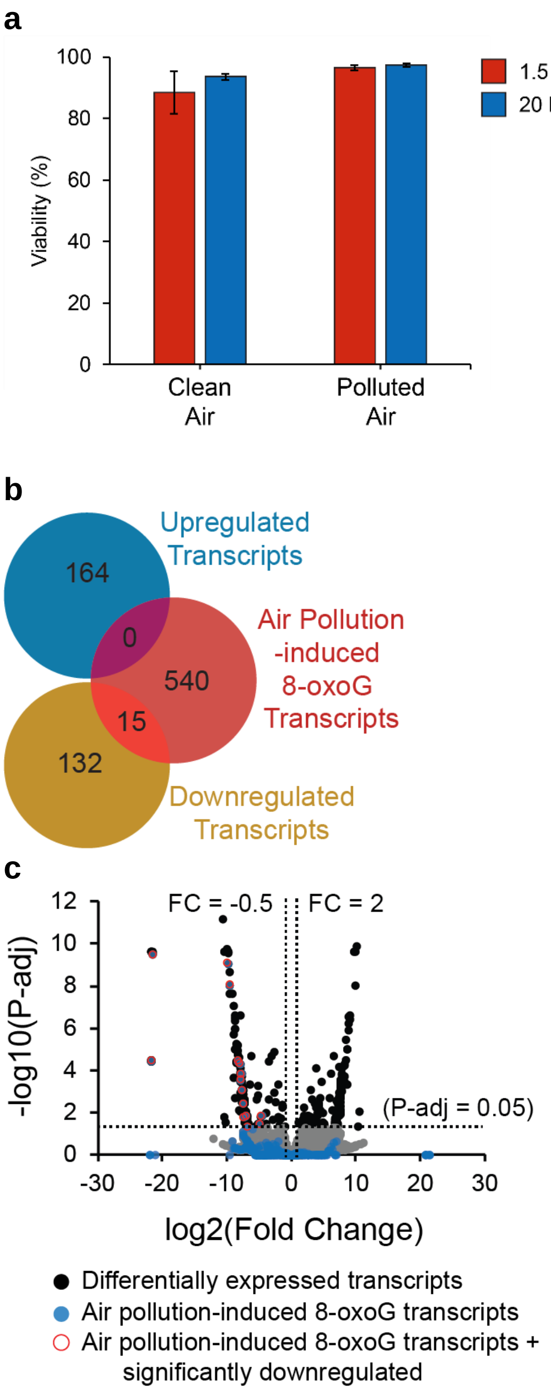


**Supplementary Fig. 10**

**a** Percentage of viable cells (at t = 1.5 h) after trypsinization of the adhered cells in the inserts, and after cell recovery (t = 20 h) determined by trypan blue dye exclusion method in an automatic viability analyzer (Vi-CELL) (n = 3 independent experiments) in cells exposed to high-levels of air pollution mixture. (B) Venn diagram shows the number of transcripts upregulated (Supplementary Data 11), downregulated (Supplementary Data 12) and 8-oxoG enriched (Supplementary Data 6) in BEAS-2B cells following high-level exposure, and the overlap with transcripts identified as prone to 8-oxoG oxidation after

exposure (Supplementary Data 13). The functional enrichment analysis of these transcripts is presented in Supplementary Data 14 and 15. (C) Volcano plot shows differential expression by comparing the input mRNA pool between air pollution vs clean air conditions at the low-level exposure. Significant expression was established with a fold change < 0.5 or > 2 with a statistical confidence of $\alpha = 0.05$.

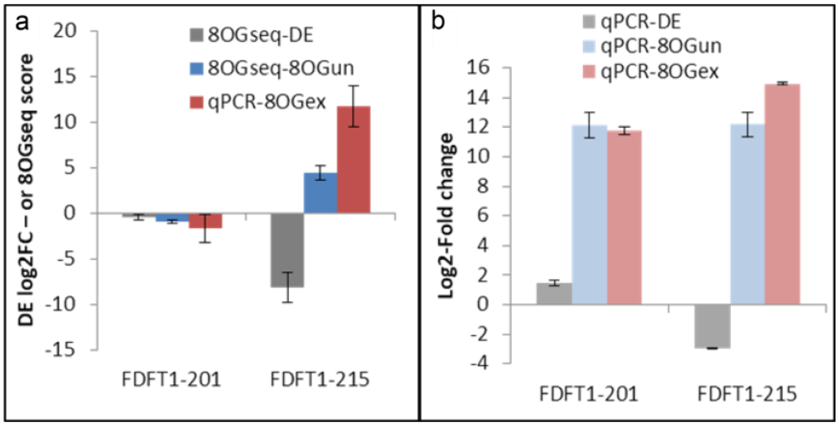


**Supplementary Fig. 11**

**a** Fold change changes for differential expression and 8-oxoG IP from BEAS-2B cells exposed at the high-level mixture as given by DESeq2. **b** Validation of the observed trends for FDFT1 was performed by qPCR quantification of 8-oxoG IP and differential expression. Importantly, the abundance patterns for the FDFT1-215 transcript identified in all three groups in the 8-oxoG analysis are replicated well by qPCR. Error bars are expressed as one standard deviation (s.d.).

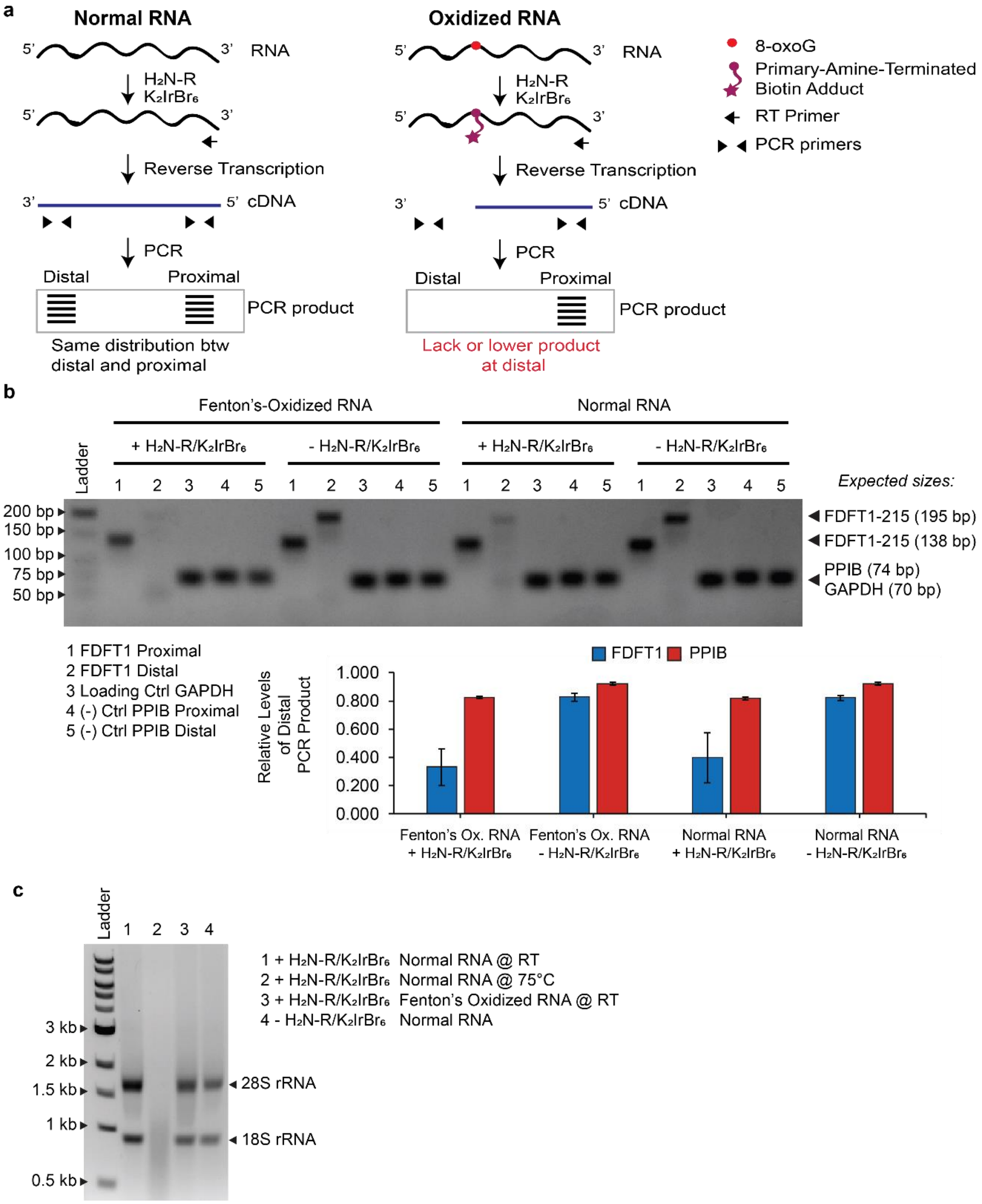


**Supplementary Fig. S12**

**a** Schematic of the reverse transcription truncation assay to validate the oxidation of the FDFT1-215 transcript via an antibody-free method. Chemical labeling of 8-oxoG with $K_2IrBr_6$ generated a covalent bond with an amine-terminated biotin with a polyethylene glycol linker (HN-R). This reaction yields a bulky moiety in 8-oxoG but not in G, which may cause reverse transcription stops. After reverse transcription of the labeled transcripts, PCR using primers near the 5' end (proximal) and the 3'end (distal) results in

accumulation of proximal products compared with the distribution of distal products. The decrease in the ratio of distal/proximal PCR products represents the relative level of 8-oxoG oxidation as compared to the control. **b** To validate the reverse transcription truncation assay with 8-oxoG chemical labeling, normal RNA extracted from BEAS-2B cells was used for a proof of concept assay. Here, we treated a fraction of the purified RNA with the Fenton's reagents to induce RNA oxidation. Normal RNA and Fenton's oxidized RNA was then chemically labeled with the biotin-terminated amine. After biotin labeling, the samples were subjected to PCR with proximal and distal primers for FDFT1-215. GAPDH and PPIB amplifications were used as loading control and negative control respectively (these transcripts were selected because they were unaffected by exposure according to our 8-oxoG RIP-seq analysis). Results indicate a decrease in the distal/proximal ratio in the Fenton's oxidized RNA compared to normal RNA when both products are biotinylated. Interestingly, non-biotinylated RNA does not stop reverse transcription as demonstrated by the constant ratio between Fenton's oxidized and normal RNA. Error bars are expressed as one standard deviation (s.d.). **c** Degradation assay of total RNA treated with the chemical labeling of 8-oxoG in 1% agarose gel electrophoresis. This assay demonstrates that chemical labeling at lower temperatures prevent degradation of the RNA as seen by the presence of intact 28S and 18S rRNA in Fenton's reaction oxidized RNA and normal RNA treated at room temperature as compared with normal RNA treated at 75 °C.  Normal RNA non-biotinylated (lane 4) was as positive control to depict intact RNA.

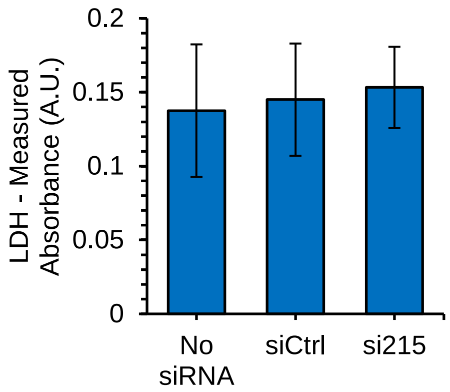


**Supplementary Fig. 13**

Cellular stress analysis by lactate dehydrogenase (LDH) release. We quantified LDH in basolateral side of BEAS-2B cells after 24-hr treatment with siRNAs by a colorimetric assay of LDH activity in the cell culture media. No statistical significance was found between conditions using t-test analysis with p-value < 0.05. Error bars are expressed as one standard deviation (s.d.).

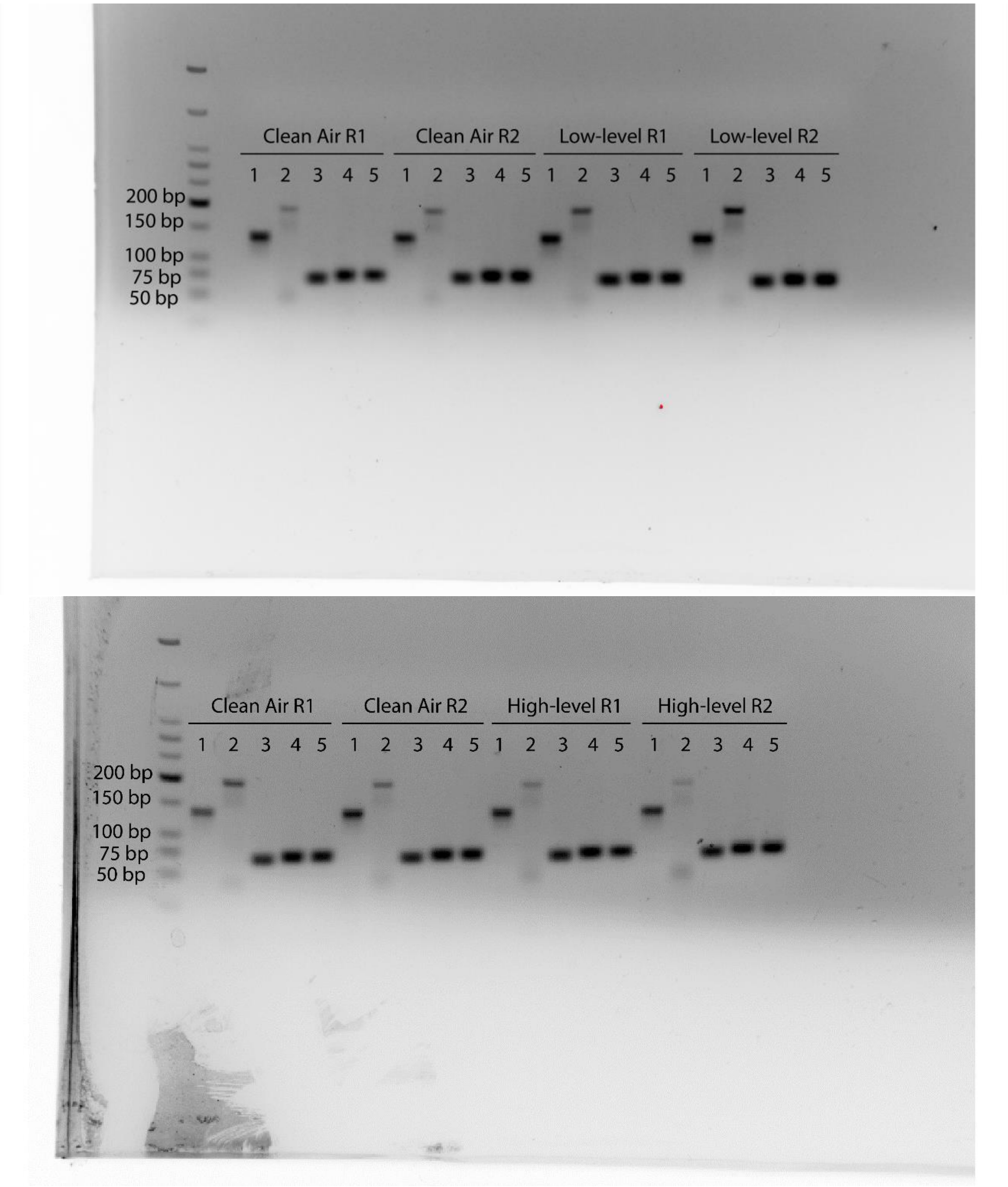


**Supplementary Figure 14**

Uncropped gel Figure 3D

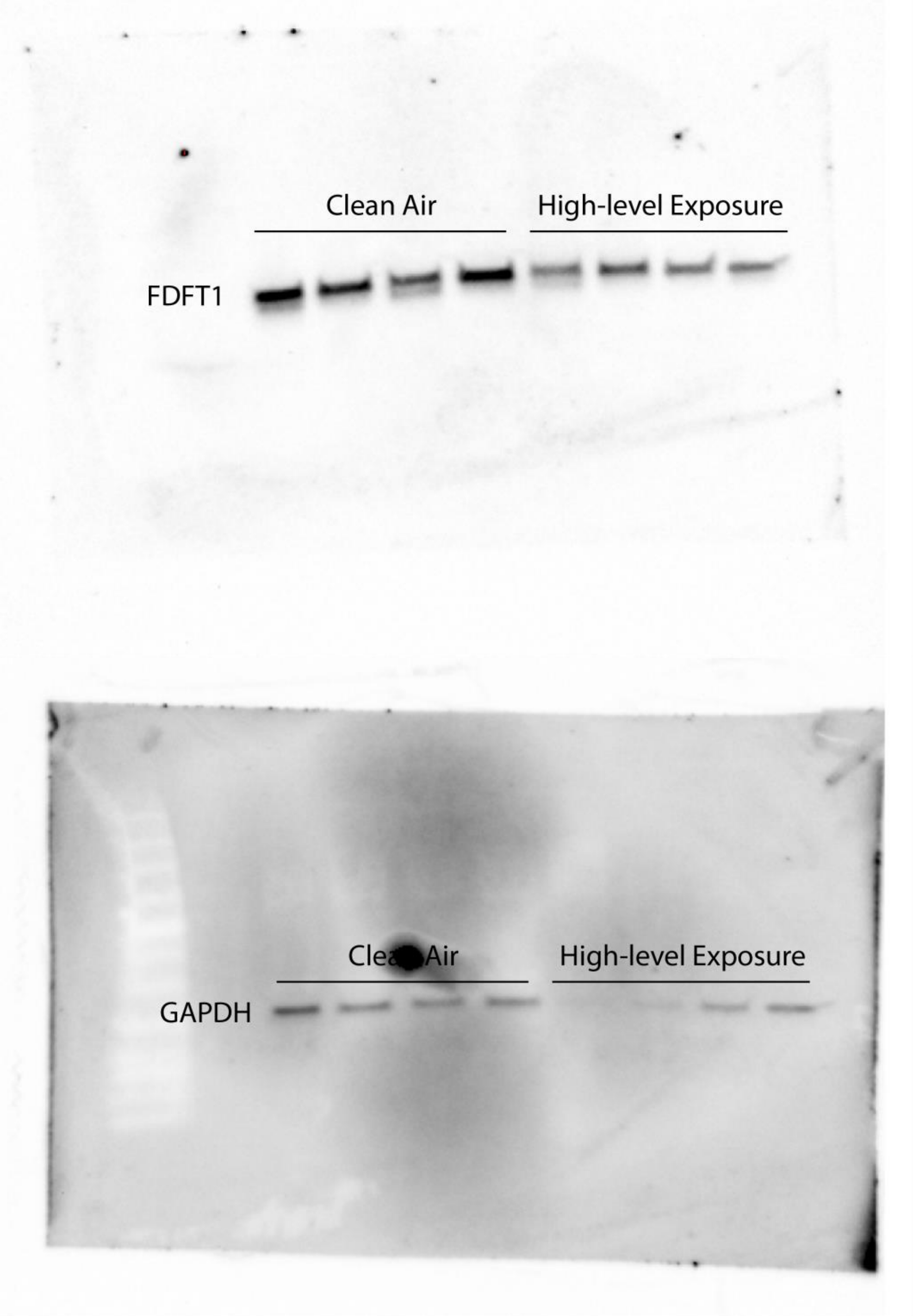
Clean Air
High-level Exposure
FDFT1
High-level Exposure
GAPDH

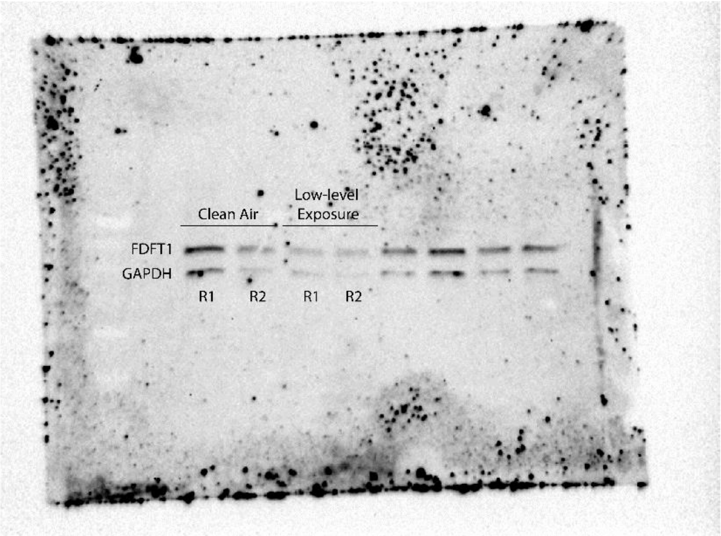


**Supplementary Figure 15**

Uncropped western blot Figure 3E

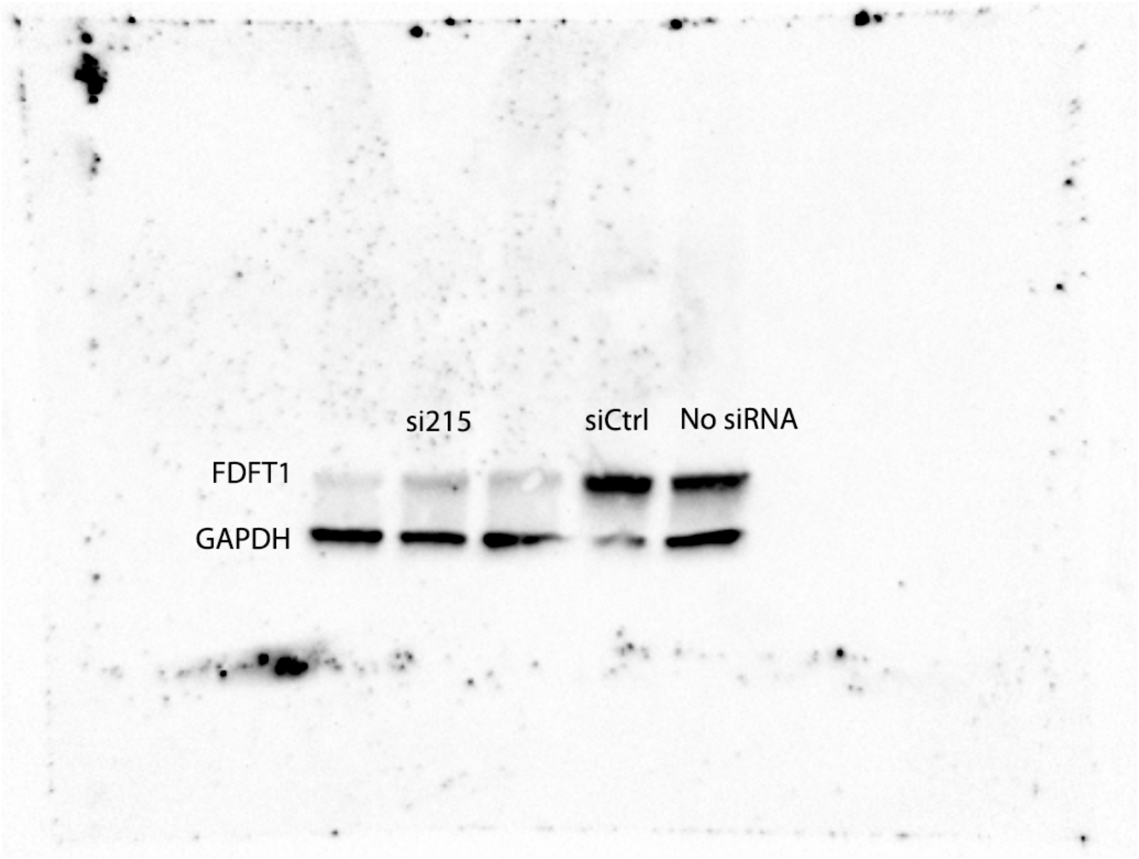


**Supplementary Figure 16**

Uncropped western blot Figure 4A

**Supplementary Table 1.** Sequences of RNA oligos for anti 8-oxoG antibody validation

| Name | Modification | Sequence 5' – 3' |
|---|---|---|
| 8-oxoG 25-mer | 8-oxoG | CGCGCGGAUCAGUXACCCAAGCGCG |
| G 25-mer | Unmodified | CGCGCGGAUCAGUCACCCAAGCGCG |
| 6x 8-oxoG random 24-mer | 8-oxoG | [NNXN]6 |
| 6x G random 24-mer | Unmodified | [NNGN]6 |
| 6x 8-oxoA random 24-mer | 8-oxoA | [NNXN]6 |
| 6x $ho^5C$ random 24-mer | $ho^5C$ | [NNXN]6 |
| 6x $ho^5U$ random 24-mer | $ho^5U$ | [NNXN]6 |
| 8-oxoG 10-mer | 8-oxoG | AXGGACUAGC |
| Unmodified 10-mer | Unmodified | AAGGACUAGC |
| f5C 10-mer | $f^5C$ | XAGGACUAGC |
| m6a 17-mer | $m^6A$ | GAACCUGXCACGUCUUA |
| A 17-mer | Unmodified | GAACCUGACACGUCUUA |

**X = Modification**

**N = A,C,G or U**

**Supplementary Table 2.** Primer sequences for reverse transcription truncation assay

| Name | Target | Primer direction | Sequence 5' - 3' | Notes |
|---|---|---|---|---|
| FDFT1-1 | FDFT1-215 | R | ACACCTGCTGGTTATTATAACAGGC | cDNA primer |
| GAPDH-1 | GAPDH | R | GTACATGACAAGGTGCGGCT | cDNA primer |
| PPIB-1 | PPIB | R | ATGGGCCTGTGGAATGTGAG | cDNA primer |
| FDFT1-4 | FDFT1-215 | F | TATTGACTTGGCCGTGCAGT | Proximal primers |
| FDFT1-10 | FDFT1-215 | R | ATGGCCATCACCTGTGGAAT | |
| FDFT1-6 | FDFT1-215 | F | ACCTACTCCACAGGTCCAGCC | Distal primers |
| FDFT1-11 | FDFT1-215 | R | ACTGCGTTGCTGGTCCATC | |
| GAPDH-2 | GAPDH | F | AGTCCCTGCCACACTCAGTC | Internal normalization control primers |
| GAPDH-3 | GAPDH | R | CAAGGGGTCTACATGGCAACT | |
| PPIB-2 | PPIB | F | AAGTTCTAGAGGGCATGGAGG | Proximal primers |
| PPIB-3 | PPIB | R | CTTCAGGGGTTTATCCCGGC | |
| PPIB-5 | PPIB | F | AAGAAGAAGGGGCCCAAAGTC | Distal primers |
| PPIB-6 | PPIB | R | ACCCGGCCTACATCTTCATCT | |
