# Supplementary material for: Post-transcriptional air pollution oxidation to the cholesterol biosynthesis pathway promotes pulmonary stress phenotypes
Source: Commun Biol. 2020 Jul 22;3:392. doi: 10.1038/s42003-020-01118-6 (PMC7376215; doi:10.1038/s42003-020-01118-6)
Supplement: Supplementary file 2 — Description of Additional Supplementary Files [file 42003_2020_1118_MOESM2_ESM.pdf]

Description of Additional Supplementary Files

File Name: Supplementary Data 1 – 15
Description: Transcripts and pathways lists underlying the 8-oxoG RIP-seq and transcriptomics analysis described in the main document and supplementary figures

Supplementary Data 1. List of significantly upregulated transcripts upon exposure at low-level mixture
Supplementary Data 2. List of significantly downregulated transcripts upon exposure at low-level mixture
Supplementary Data 3. List of air pollution-induced oxidized transcripts at low-level mixture
Supplementary Data 4. List of enriched KEGG pathways in significantly oxidized transcripts upon exposure at low-level mixture
Supplementary Data 5. List of air pollution-induced oxidized transcripts with significant downregulation at low-level mixture
Supplementary Data 6. List of air pollution-induced oxidized transcripts at high-level mixture
Supplementary Data 7. List of overlapped air pollution-induced oxidized transcripts between the low-level and high-level exposures
Supplementary Data 8. List of enriched KEGG pathways in significantly oxidized transcripts upon exposure at high-level mixture
Supplementary Data 9. List of enriched KEGG pathways in upregulated transcripts upon exposure at low-level mixture
Supplementary Data 10. List of enriched KEGG pathways in downregulated transcripts upon exposure at low-level mixture
Supplementary Data 11. List of significantly upregulated transcripts upon exposure at high-level mixture
Supplementary Data 12. List of significantly downregulated transcripts upon exposure at high-level mixture
Supplementary Data 13. List of air pollution-induced oxidized transcripts with significant downregulation at high-level mixture
Supplementary Data 14. List of enriched KEGG pathways in upregulated transcripts upon exposure at high-level mixture
Supplementary Data 15. List of enriched KEGG pathways in downregulated transcripts upon exposure at high-level mixture

File Name: Supplementary Data 16
Description: All source of data underlying the graphs presented in the main figures
