# Supplementary material for: Post-transcriptional air pollution oxidation to the cholesterol biosynthesis pathway promotes pulmonary stress phenotypes
Source: Commun Biol. 2020 Jul 22;3:392. doi: 10.1038/s42003-020-01118-6 (PMC7376215; doi:10.1038/s42003-020-01118-6)
Supplement: Supplementary file 5 — Reporting Summary [file 42003_2020_1118_MOESM5_ESM.pdf]

nature research

Corresponding author(s): Lydia Contreras
Last updated by author(s): Jun 15, 2020

Reporting Summary

Nature Research wishes to improve the reproducibility of the work that we publish. This form provides structure for consistency and transparency in reporting. For further information on Nature Research policies, see Authors & Referees and the Editorial Policy Checklist.

Statistics

For all statistical analyses, confirm that the following items are present in the figure legend, table legend, main text, or Methods section.

n/a	Confirmed	
☐	☒	The exact sample size (n) for each experimental group/condition, given as a discrete number and unit of measurement
☐	☒	A statement on whether measurements were taken from distinct samples or whether the same sample was measured repeatedly
☐	☒	The statistical test(s) used AND whether they are one- or two-sided<br>*Only common tests should be described solely by name; describe more complex techniques in the Methods section.*
☒	☐	A description of all covariates tested
☐	☒	A description of any assumptions or corrections, such as tests of normality and adjustment for multiple comparisons
☐	☒	A full description of the statistical parameters including central tendency (e.g. means) or other basic estimates (e.g. regression coefficient) AND variation (e.g. standard deviation) or associated estimates of uncertainty (e.g. confidence intervals)
☐	☒	For null hypothesis testing, the test statistic (e.g. F, t, r) with confidence intervals, effect sizes, degrees of freedom and P value noted<br>*Give P values as exact values whenever suitable.*
☒	☐	For Bayesian analysis, information on the choice of priors and Markov chain Monte Carlo settings
☒	☐	For hierarchical and complex designs, identification of the appropriate level for tests and full reporting of outcomes
☒	☐	Estimates of effect sizes (e.g. Cohen's d, Pearson's r), indicating how they were calculated

Our web collection on statistics for biologists contains articles on many of the points above.

Software and code

Policy information about availability of computer code

Data collection	Vi-Cell XR 2.03 for collection of cell density and viability<br>Nanodrop 2000/2000C v 13.1 for RNA concentration<br>Gen5. 2.04 for collection of absorbance for cytotoxicity analysis, ELISA and cholesterol<br>ImageLab V. 5.2.1 build 11 for collection of blotting signal and RNA staining<br>ZEN 2.5 for collection of confocal microscopy images<br>AxioVision SE64 V 4.9.1 for collection of widefield microscopy images
Data analysis	Microsoft Excel (Microsoft Office 365 ProPlus) for data organization, analysis and plotting of graphs<br>Prism 8 for plotting graphs<br>FastQC Version 0.11.9 for generation of quality check reports of the transcriptomics data<br>cutadapt 1.14 for trimming adapters<br>STAR 2.6.0c for read mapping<br>RSEM 1.3.1 was used to estimate transcript abundances<br>DESeq2 in R version 3.6.1 for differential expression and statistical analysis<br>CLIQS (TotalLab) v1.1 for gel analysis<br>Fiji/ImageJ (64-bit windows) for characterization of cell morphology<br>Fibriltool plugin in Fiji (2014) for measurement of fibril organization<br>Microsoft Excel and Prims 8 for statistical analysis

For manuscripts utilizing custom algorithms or software that are central to the research but not yet described in published literature, software must be made available to editors/reviewers. We strongly encourage code deposition in a community repository (e.g. GitHub). See the Nature Research guidelines for submitting code & software for further information.

Data

Policy information about availability of data

All manuscripts must include a data availability statement. This statement should provide the following information, where applicable:

- Accession codes, unique identifiers, or web links for publicly available datasets
- A list of figures that have associated raw data
- A description of any restrictions on data availability

RNA-sequencing datasets have been deposited in NCBI GEO under accession number GSE137019.

Field-specific reporting

Please select the one below that is the best fit for your research. If you are not sure, read the appropriate sections before making your selection.

☒ Life sciences ☐ Behavioural & social sciences ☐ Ecological, evolutionary & environmental sciences

For a reference copy of the document with all sections, see nature.com/documents/nr-reporting-summary-flat.pdf

Life sciences study design

All studies must disclose on these points even when the disclosure is negative.

Sample size	All the experiments were conducted in duplicates or triplicates. To generate RNA samples for RNA sequencing, we conducted three independent exposures in different days alongside clean air controls. Before preparing samples for 8-oxoG immunoprecipitation, we confirmed expected trends on 8-oxoG levels and cytotoxicity in triplicates. RNA extractions from independent cell culture were analyzed by Bioanalyzer before conducting 8-oxoG immunoprecipitation. Transcriptomic analysis was conducted in duplicates from two independent exposures. To generate RNA and lysate for validation of the oxidation in FDFT1, western blotting, cholesterol analysis and knockout experiments, we conducted two independent exposures in different days.
Data exclusions	Data was excluded in the microscopy analysis when outliers deviated remarkably from other observations in the sample. We accepted exclusions for this analysis because the sample size was at least 10 cells for each experiment.
Replication	We conducted at least two technical replicates for all the experiments. ELISA experiments that had more variability were conducted with three replicates and two dilutions. Replications were within 10% error rate or less than one standard deviation in all the experiments.
Randomization	Randomization of samples for microscopy analysis was conducted using a probability function in excel. Other experiments were not randomized because it is not applicable for the number of samples (duplicates or triplicates) and setup of the experiments
Blinding	According to blinding guidelines this test is not applicable for cell line studies

Reporting for specific materials, systems and methods

We require information from authors about some types of materials, experimental systems and methods used in many studies. Here, indicate whether each material, system or method listed is relevant to your study. If you are not sure if a list item applies to your research, read the appropriate section before selecting a response.

Materials & experimental systems

n/a	Involved in the study	
☐	☒	Antibodies
☐	☒	Eukaryotic cell lines
☒	☐	Palaeontology
☒	☐	Animals and other organisms
☒	☐	Human research participants
☒	☐	Clinical data

Methods

n/a	Involved in the study	
☒	☐	ChIP-seq
☒	☐	Flow cytometry
☒	☐	MRI-based neuroimaging

Antibodies

Antibodies used	8-oxo-7,8-dihydroguanosine (8-oxoG) monoclonal antibody (0.5 mg/ml, Clone 15A3, Trevigen)
Validation	Antibody was validated via dot blot analysis. All RNA oligomers used to test the specificity of the commercially available 8-oxoG antibody (clone 15A3) employed in 8-oxoG RIP-seq are listed in Table S1A and were synthesized by GeneLink. Serial 2-fold dilutions of each oligo were denatured and 5 µl was spotted on the hybond-N+ nylon membrane (GE Healthcare) followed by UV-crosslinked at 120,000 µJ/cm for 60 seconds. The membrane was blocked with 5% Bovine Serum Albumin (BSA; Fisher Scientific) in 1X PBS (pH 7.4, VWR) containing 0.05% Tween 20 (VWR) overnight at 4°C. After extensive washing, it was incubated

at 4 °C in 1% BSA in 1X PBS with the addition of anti-8-oxoG antibody (clone 15A3, Trevigen) used at 1:400 dilution. Following extensive washing, the membrane was incubated at room temperature for 1 hour with anti-mouse IgG H&L HRP conjugate (W4021, Promega) secondary antibody diluted 1:2,500 in in 1% BSA in 1X PBS. Chemiluminescent detection was conducted on a ChemiDoc XRS+ imaging system (Biorad) and quantification of the band's intensity with CLIQS (TotalLab).

Eukaryotic cell lines

Policy information about cell lines

Cell line source(s)	BEAS-2B cell line from ATCC
Authentication	Cell morphology characterization via microscopy
Mycoplasma contamination	Cells were not tested for mycoplasma contamination
Commonly misidentified lines (See ICLAC register)	N/A
